# Supplementary material for: In-situ plasmonic tracking oxygen evolution reveals multistage oxygen diffusion and accumulating inhibition
Source: Nat Commun. 2021 Apr 12;12:2164. doi: 10.1038/s41467-021-22434-3 (PMC8041856; doi:10.1038/s41467-021-22434-3)
Supplement: Supplementary file 1 — Supplementary Information [file 41467_2021_22434_MOESM1_ESM.pdf]

Supplementary Information For

In-Situ Plasmonic Tracking Oxygen Evolution Reveals Multistage Oxygen Diffusion and Accumulating Inhibition

Jun-Gang Wang,[1] Lifang Shi,[2] Yingying Su,[1, 3] Liwei Liu,[2] Zhenzhong Yang,[4] Rong Huang,[4] Jing Xie,[5] Yang Tian,[1] and Di Li*[1,3]

[1] School of Chemistry and Molecular Engineering, East China Normal University, Shanghai 200241, China

[2] Institute of Optics and Electronics, Chinese Academy of Sciences, Chengdu 610209, China

[3] Key Laboratory of Bioorganic Phosphorus Chemistry & Chemical Biology, Department of Chemistry, Tsinghua University, Beijing, 10084, China

[4] Key Laboratory of Polar Materials and Devices (MOE) and Department of Electronics, East China Normal University, Shanghai 200241, China

[5] Institute of Microelectronics, Chinese Academy of Sciences, Beijing 100029, China

Supplementary Note 1: Experimental Section

Chemicals

Copper sulfate ($CuSO_4$, ≥ 99.5 %) was purchased from J&K Scientific Ltd. (Shanghai, China). Potassium hydroxide (KOH, ≥ 85.0 %), sodium perchlorate monohydrate ($NaClO_4$ H_2O, ≥ 99.0 %), potassium ferricyanide ($K_3Fe(CN)_6$, ≥ 99.5 %), Acetone (analytical reagent), isopropanol (analytical reagent) and ethanol (analytical reagent) and hydrofluoric acid (analytical reagent) were purchased from Sinopharm Chemical Reagent Co., Ltd. (Shanghai, China). All reagents were of analytical grade and were used as received without any further purification. The solution was prepared using deionized water. Deionized water filtered by a Milli-Q system at a resistivity of > 18.2 MΩ cm and then re-filtered by 200 nm membrane filters was used throughout the experiments. ITO-coated glass (sheet resistance 20−30 $\Omega\ sp^{-1}$; 1 mm) was purchased from Shenzhen Laibao Technology Co. Ltd. (Shenzhen China).

Electrochemical Deposition of Cu NPs on ITO

The ITO-coated glass was firstly cleansed with ethanol, and washed successively in acetone, isopropanol and water with at least 30 min under sonication, and dried under a flow of nitrogen gas. Then the ITO electrodes were incubated in 0.1 M HF for 30 s and rinsed three times with copious deionized water. Subsequently, the electrodes were treated with O_2 plasmon for 5 min (Harrick, PDC-32G-II, USA).[1-2] A saturated calomel reference electrode (SCE) was used as reference electrode, and a Pt wire was used as the counter electrode for deposition of Cu seeds and subsequent growth of Cu NPs on the ITO electrode.

For the electrodeposition of Cu NPs, a deposition solution containing 1.0 mM $CuSO_4$ and 0.10 M $NaClO_4$ was used. First, a potential step from 0.20 to - 0.60 V was applied on the deposition solution, then the open circuit potential of the ITO working electrode was changed from 0.20 to - 0.01 V (*vs.* SCE). The morphology of the Cu seeds and bare ITO electrode was shown in the Supplementary Figure 6. Second, the as prepared Cu seeds on ITO electrode (abbreviated to Cu seeds/ITO electrode) was served as the working electrode for subsequent growth of Cu NPs in the same electrochemical cell. The growth of Cu NPs was performed by a cyclic voltammetry during a dynamic potential range from – 0.10 to – 0.30 V (*vs.* SCE) with different deposition cycles, at a scan rate of 0.05 V s^{-1}. Then, the as-prepared electrode

(abbreviated to Cu NPs/ITO electrode) was removed from the deposition solution and rinsed copiously with deionized water. Last, the Cu NPs/ITO electrode was dried under a gentle stream of nitrogen before its utilization for gas evolution reaction.

Simulations

Full-wave electromagnetic simulation was performed on a commercial software (LUMERICAL, FDTD Solution) and finite element models (FEM) simulation was carried out by COMSOL Multiphysics 5.3a software (COMSOL Inc.) on a high-performance desktop workstation (16 G RAM). For FDTD simulations, the perfectly matched layers were used along the propagation directions and the spatial grids' size in all axes was set to 2.0 nm. Consider the proposed model, the complex refractive index of Cu_2O, CuO and $Cu(OH)_2$ were taken from literatures.[3-4] Both magnetic- and electric- field enhancement maps under different excitation wavelength were calculated in FDTD using frequency domain field. The extinction spectra of proposed configurations were conducted using a regular plane-wave with a pulse length of 2.6 fs. The power monitor is located in a plane 14 nm away from the metal film. In order to eliminate the contribution from random dispersed particles induced over-evaluation on the gap distance, a gap distance 26 nm for Cu NPs/ITO (150 cycles) was adopt in simulation (Supplementary Figure 8). We adopted two strategies to approximately estimate the influence of oxygen accumulation on the optical properties based on the individual unit cell dimer-on-film structure shown in Supplementary Figure 8.

(1) As oxygen evolution, the refractive index of the electrolyte (1.33) located at interfacial region could gradually decrease due to the accumulation of oxygen molecules (refractive index: 1.00).[5] Because the limitation in precise identification of absolute oxygen centration close to the electrode surface, it is very hard and impossible to acquire the dynamic hybrid *refractive index* of interfacial electrolyte (including solved oxygen) during dynamic potential scan. To qualitatively evaluate the influence of oxygen accumulation, we rationally set the refractive index of environment from 1.33 to 1.06 in simulating the dynamic environment in potential range from + 0.20 V to + 0.90 V then to + 0.67 V (Region III corresponding to 130 s to 220 s in time scale); To further evaluate subsequent oxygen spontaneous and accelerated diffusion processes, the refractive index of environment was

rationally set from 1.06 to 1.20 in potential range from + 0.67 V to – 0.35 V (Region IV corresponding to 220 s to 319 s in time scale) and from 1.20 to 1.33 in potential range from – 0.35 V to – 0.63 V (Region V corresponding to 319 s to 365 s in time scale), respectively (Supplementary Figure 28 a, b).

(2) We then considered an extreme condition, where oxygen molecules are accumulated on the electrode surface to form an oxygen shell around Cu NPs, this enables to exclude surrounding aqueous electrolyte solution and to decrease the refractive index of environment. To qualitatively evaluate the influence of oxygen accumulation, we rationally set the thickness of the oxygen shell from 0 nm to 120 nm to simulate the dynamic environment in the potential range from + 0.20 V to + 0.90 V then to + 0.67 V (Region III corresponding to 130 s to 220 s in time scale); To further evaluate subsequent oxygen spontaneous and accelerated diffusion processes, the thickness of oxygen layer was rationally set from 120 nm to 85 nm in the potential range from + 0.67 V to – 0.35 V (Region IV corresponding to 220 s to 319 s in time scale) and from 85 nm to 0 nm in potential range from – 0.35 V to – 0.63 V (Region V corresponding to 319 s to 365 s in time scale), respectively (Supplementary Figure 28 c, d).

Despite all these efforts, it should be noted that the dynamic of oxygen diffusion at the EEIs is far more complicated than what we proposed here. Our simulation results only provide a qualitative evaluation of interfacial processes, a more precise description of the dynamic interfacial electrochemical reactions should consider electric field interference, build more complicated models and adopt density functional methods or molecular dynamics simulations.

Supplementary Note 2：Electrochemical Seed-Mediated Deposition of Cu NPs on ITO Electrode

In classical nucleation theory, nucleation of a new solid phase on a heterogeneous interface has to overcome a free energy barrier related to the thermodynamic costs for forming a critical cluster of atoms.[6] During electrodeposition, this nucleation energy barrier can be effectively regulated by changing the local electrochemical supersaturation at the electrode-electrolyte interface through adjusting the applied overpotential on the reduction reaction.[7] The driving

force induced by reaction, charge transfer and diffusion as well as crystallization overpotential work together on the electrodeposition process.[8] Considering the difficulty in identification of each overpotential contribution, here we referred to nucleation (η_n) and growth (η_g) overpotential for explicit describing Cu electrodeposition (Supplementary Figure 1). During initial nucleation stage, an essential prerequisite for nucleation of Cu embryos was a sufficient overpotential, which enabled to surmount the large free energy barrier (ΔG_n). After nucleation occurs, attributed to the lowering free energy barrier (ΔG_g) for the growth of Cu seeds, a lower overpotential was competent in subsequent growth process. Because of the lower free energy barrier, it was more thermodynamic favorable to introduce a Cu adatom on a Cu nuclei interface than re-nucleation on a non-homologous heterogeneous interface. Taken together the difference of the free energy barrier in both nucleation and growth, an electrochemical seed-mediated strategy enabled the efficient adjustment the Cu electrodeposition (Supplementary Figure 1, a and b).

To construct the plasmonic-enhanced system, the copper seeds were firstly deposited on ITO electrode at a fixed polarization nucleation pulse (Figure 1, a, i and Supplementary Figure 1, c). With the prior deposition of copper seeds, a positive shift *ca.* 20 mV (*vs.* SCE) of the reduction potential for copper ions reduction to copper reduced from – 0.12 to – 0.10 V (*vs.* SCE), indicating the elimination of the activation energy for Cu^{2+} reduction on the electrode (Supplementary Figure 2). Hence, it provided the discrete preferential nucleation sites for subsequent copper deposition, avoiding the random heterogeneous nucleation. For achieving high copper seeds coverage, the cathodic polarization potential was optimized without the disturbance from side reaction, such as hydrogen evolution reaction. The detailed electrochemical and time-of-flight secondary ion mass spectrometry analysis was shown in Supplementary Figure 3-4. Supplementary Figure 3 indicated that the amount of charge during the nucleation increased drastically accompanied by the Cu coverages reduction beyond – 0.60 V *vs.* SCE. It should be noted that the immoderate enhancement of polarization overpotential resulted in the co-electrolysis of Cu electrodeposition and hydrogen evolution that required extra amount charge to complete the nucleation step. Specifically, hydrogen evolution competed with the Cu nucleation and restrained the diffusion of the Cu^{2+} to the electrode surface, as indicated by the decreased diffusion coefficient of Cu^{2+} from 2.20×10^{6} cm^2 s^{-1} at –

0.60 V to 1.98 $\times10^6$ cm^2 s^{-1} at – 0.90 V (Supplementary Figure 4). In order to eliminate the co-electrocatalysis of hydrogen evolution and increase the efficiency of Cu electrodeposition, a polarization potential at – 0.6 V *vs.* SCE was chosen for the initial Cu nucleation and followed by a relative lower polarization potential window from – 0.10 to – 0.30 V *vs.* SCE for subsequent growth of the presence Cu nuclei (Supplementary Figure 5).

The diffusion coefficient of Cu^{2+} during electrochemical deposition condition was acquired from Cottrell equation. In brief, the bare ITO electrode was submerged in the solution containing 1 mM $CuSO_4$ and 0.1 M $NaClO_4$ with a Pt wire counter electrode and a SCE reference electrode (Supplementary Figure 4). The current transients were recorded for electrodeposition of Cu NPs by applying a potential step from 0.20 V to various termination potential (− 0.50, − 0.60, − 0.70, − 0.80 and − 0.90 V *vs.* SCE). The current decay was linear with $t^{-1/2}$, and it indicated a planar diffusion regime emerged caused by the overlapping of growing hemispherical diffusion layers, which provided mass transport for nanocrystal growth in the process of copper nucleation.[9-10] The diffusion coefficient could be obtained from the slop of i versus $t^{-1/2}$ plot based on the Cottrell equation:[11]

$$i = nFAD_{Cu^{2+}}{}^{1/2}c_{Cu^{2+}}\pi^{-1/2}t^{-1/2} \quad (2\text{-}1)$$

where n is the number of transferred electrons (n = 2), F is the Faraday's constant (96485 C mol^{-1}), D_{Cu2+} and C_{Cu2+} is the diffusion coefficient and the bulk concentration of Cu^{2+} (1 mM), A is the geometric area of the bare ITO electrode 1.5 cm^2 (width 7.5 mm and length 20 mm). The calculated diffusion coefficient for copper electrodeposition on a bare ITO electrode under cathodic polarization potential (− 0.60 V) was 2.2×10^{-6} cm^2 s^{-1}, which was on the same magnitude compared to the diffusion coefficient of Cu^{2+} in water (5.0 $\times10^{-6}$ cm^2 s^{-1})[12] and the polarographic diffusion coefficient of copper ions (7.2 $\times10^{-6}$ cm^2 s^{-1}).[13] Note that no remarkable initial current spine caused by the charging of the double electric layer was observed (Supplementary Figure 3, a-e).

It could be concluded that the growth rate of copper nuclei was extremely rapid. The rising portion of the transient current was not experimentally accessible under our conditions, thus the estimation of the nucleation mechanism (i.e., instantaneous or progressive) of copper nanoparticles was not possible from the transient current curves.[14] However, it was reasonable

to conjecture that the electrochemical reduction process of Cu^{2+} described here experienced an instantaneous nucleation process on a bare ITO electrodes based on the fact that no noteworthy charging phase was observed.[15] The electrochemical formed copper seeds exhibited higher coverage and favorable mono-dispersity than progressive nucleation, which could avoid seriously coupling of the diffusion layer of the adjacent copper nanoparticles and mitigate deteriorative inhomogeneity of the particle size.

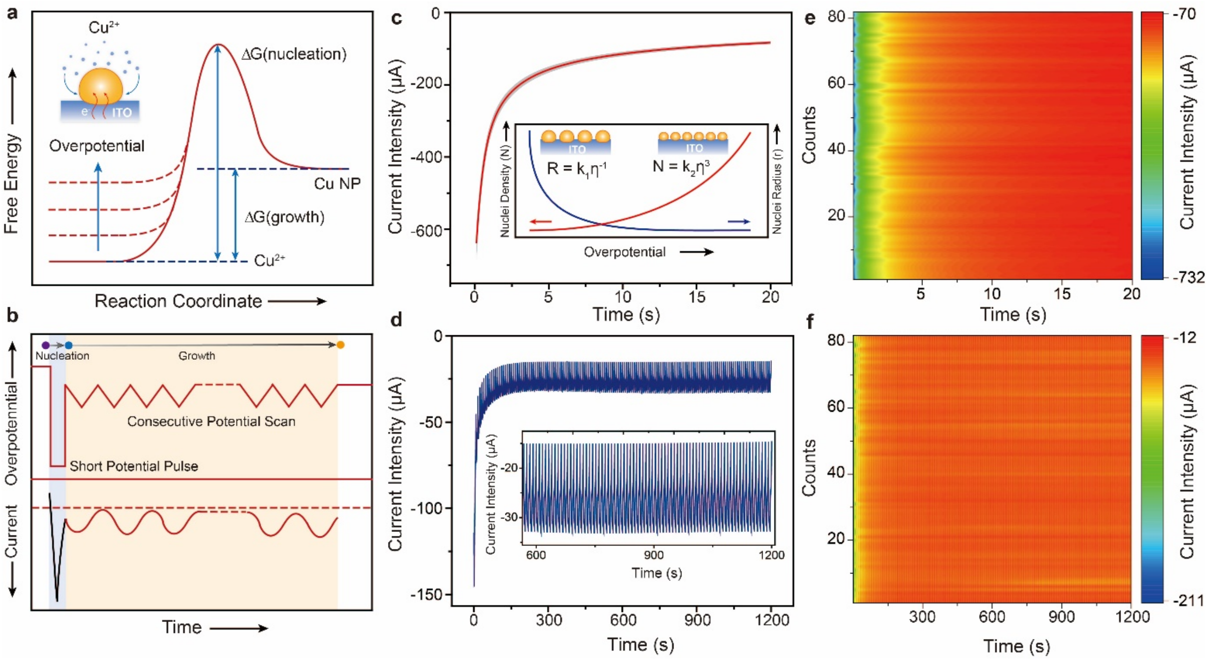

Supplementary Figure 1. (a) Schematic illustration of the free energy evolution during electrodeposition of Cu NPs. The nucleation energy barrier of Cu seeds decreased with increasing of the applied overpotential. (b) Schematic of the electrochemical seed-mediated growth of Cu NPs on ITO electrode. This strategy had two main steps: firstly, the seeding step under enhanced cathodic polarization; secondly, the growth step with lower cathodic polarization. The schematic of overpotential and relative current for electrochemical deposition of Cu NPs were shown in top and bottom panel, respectively. (c) The experimental chronoamperometry profile for the seeding step at − 0.60 V (*vs.* SCE), 20 s. The dark region represented the standard error for five independent measurements. Inset showed schematic of the dependence of the nucleation density, nuclei radius on the applied overpotential for Cu deposition. (d) The experimental current profile for the growth step within a potential scanned ranging from − 0.10 to − 0.30 V (*vs.* SCE), at a scan rate of 0.05 V s^{-1}, 150 cycles. Inset showed the enlarged view of the current profile. (e) The contour plots of chronoamperometry profiles for the seeding step within independent 82 measurements. (f) The contour plots of relative current profiles for the growth step within independent 82 measurements. Both homogeneity and stability of the electrochemical seed-mediated growth of Cu NPs enabled reproducible fabrication of Cu NPs/ITO electrodes and circumvented the challenge of handling nanoparticles that were easily aggregated.

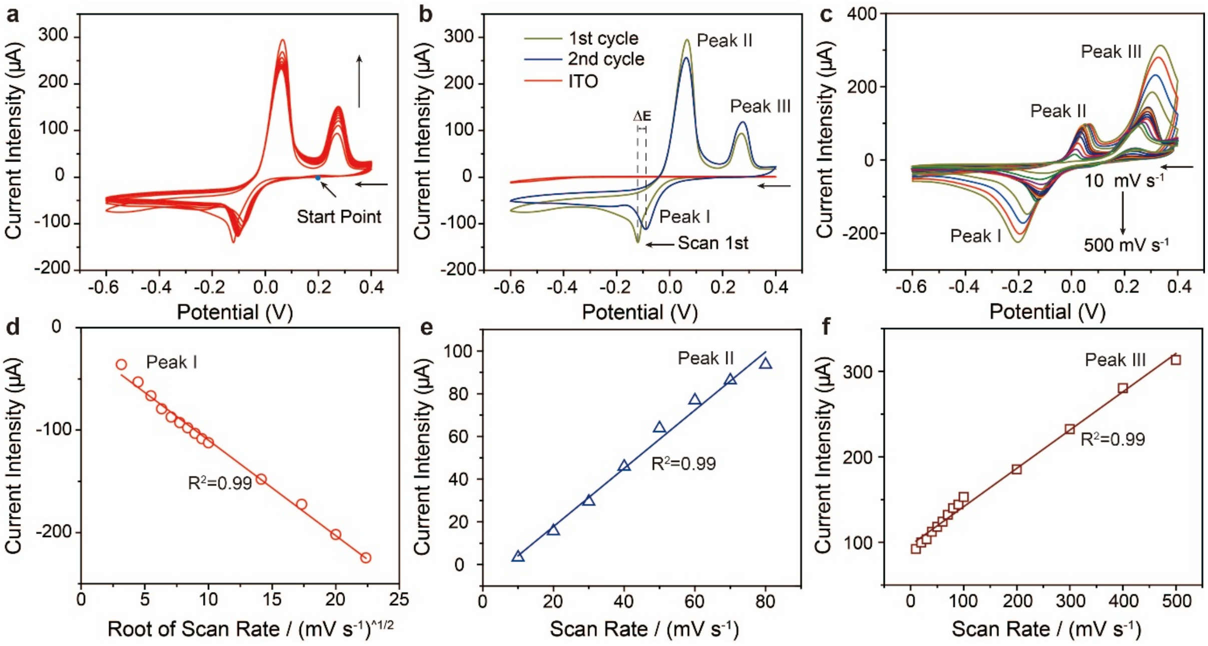

Supplementary Figure 2. (a) Cylic voltammograms (CV) for electrodeposition of Cu nanoparticles on a bare ITO electrode in 1.0 mM $CuSO_4$ and 0.10 M $NaClO_4$ at a potential range from − 0.60 to 0.40 V (*vs.* SCE), at a scan rate of 0.05 V s^{-1}. The start potential located at 0.20 V. Owing to the Cu nanoparticles deposited on the ITO electrode, the increased electrode surface area contributed to the gradual enhancement of Cu oxidation peak current. (b) The comparison of CV curves between first cycle (green line) and the second cycle (blue line) in 1.0 mM $CuSO_4$ and 0.10 M $NaClO_4$, at a scan rate of 0.05 V s^{-1}. The control cyclic voltammetry of bare ITO electrode (red line) in 0.10 M $NaClO_4$, at a scan rate of 0.05 V s^{-1}. The presence of Cu nanoparticles on the ITO electrode enabled the decrease of the nucleation energy barrier which facilitated the growth of Cu nanoparticles. (c) Scan rate dependence of CV curves for a bare ITO electrode in 1.0 mM $CuSO_4$ and 0.10 M $NaClO_4$, at various scan rates 0.01, 0.02, 0.03, 0.04, 0.05, 0.06, 0.07, 0.08, 0.09, 0.10, 0.20, 0.30, 0.40 and 0.50 V s^{-1}. The cathodic peak I was assigned to the reduction of Cu^{2+} to Cu. The other two anodic peak II and III was ascribed to the oxidation of Cu to Cu^{+} and Cu to Cu^{2+}, respectively. The dependence of the peak currents of peak I on the square root of the scan rate, revealing a diffusion-controlled kinetic process during reduction of Cu^{2+}. Meanwhile, the dependence of the peak currents of peak II (e) and III (f) on scan rate. It indicated that both the anodic processes were involved in surface reaction-controlled manner.

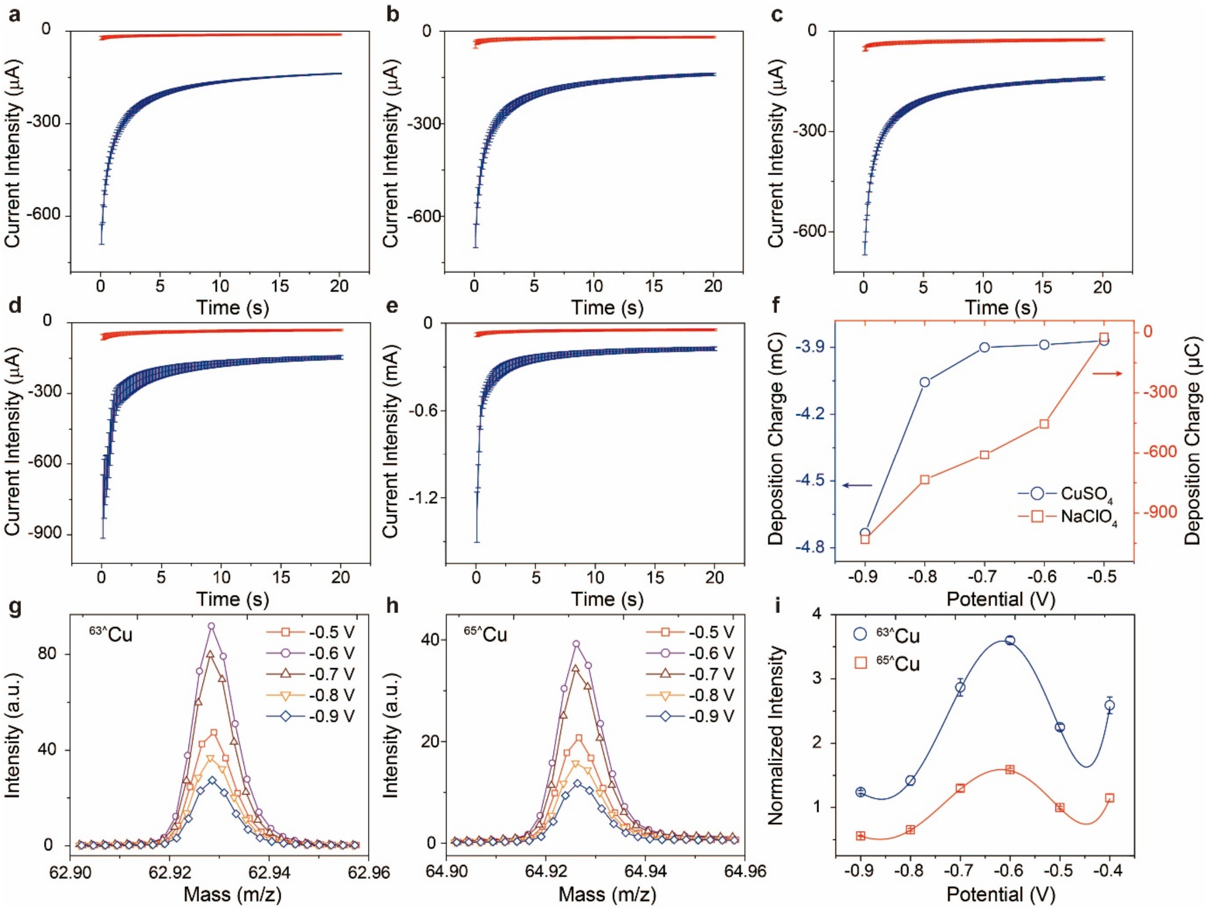

Supplementary Figure 3. Transient current recorded during a potential step from 0.20 to − 0.50 V (a), − 0.60 V (b), − 0.70 V (c), − 0.80 V (d) and − 0.90 V (e) *vs* SCE for a bare ITO electrode in 1.0 mM $CuSO_4$ and 0.10 M $NaClO_4$ (blue line) and 0.10 M $NaClO_4$ (red line), respectively. The error bar represented the standard error within three independent measurements. (f) The dependence of deposition charge during chronoamperometry measurements on the applied cathodic polarized potential in 1.0 mM $CuSO_4$ and 0.10 M $NaClO_4$ (blue circle) and 0.10 M $NaClO_4$ (red square), respectively. The dependence of the isotopic mass peak intensity of $^{63\wedge}Cu$ (g) and $^{65\wedge}Cu$ (h) on the applied cathodic polarized potential. (i) The comparison between the mass intensity of $^{63\wedge}Cu$ and $^{65\wedge}Cu$ on the applied cathodic polarized potential. The error bar represented the standard error within three independent measurements.

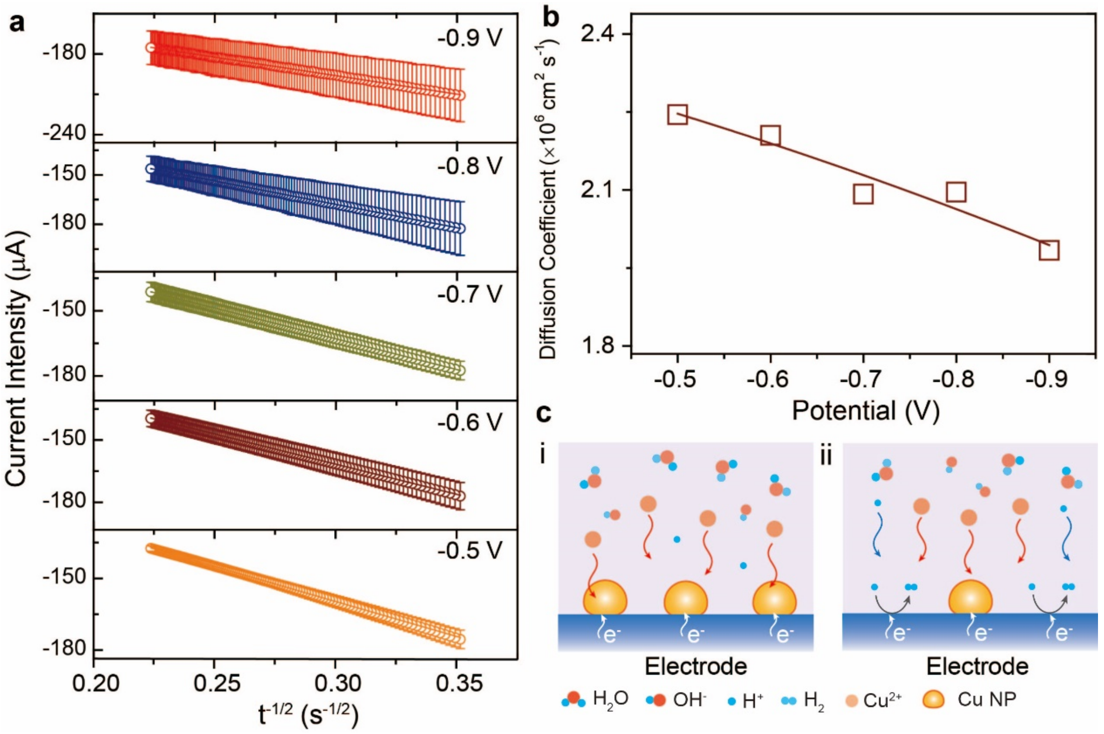

Supplementary Figure 4. (a) The plots of transient current as a function of $t^{-1/2}$ between 8 and 20 s after potential step from 0.20 V to various terminal potential (− 0.50, − 0.60, − 0.70, − 0.80 and − 0.90 *vs.* SCE) applied on the ITO electrode in 1.0 mM $CuSO_4$ and 0.10 M $NaClO_4$. The error bar represented the standard error within three independent measurements. (b) The diffusion coefficient of Cu^{2+} under various cathodic polarization was evaluated by using the extracted slopes of linear fitting lines shown in (a) based on Cottrell equation. The fitting line of the diffusion coefficient was used to visualize the trends. (c) Schematic of the diffusion of Cu^{2+} and subsequent cathodic nucleation of Cu NPs on the electrode surface under appropriate cathodic polarization (i). With the enhancement of cathodic polarization, the co-reduction occurrence of both Cu^{2+} and H^+ on the electrode-electrolyte interface (ii).

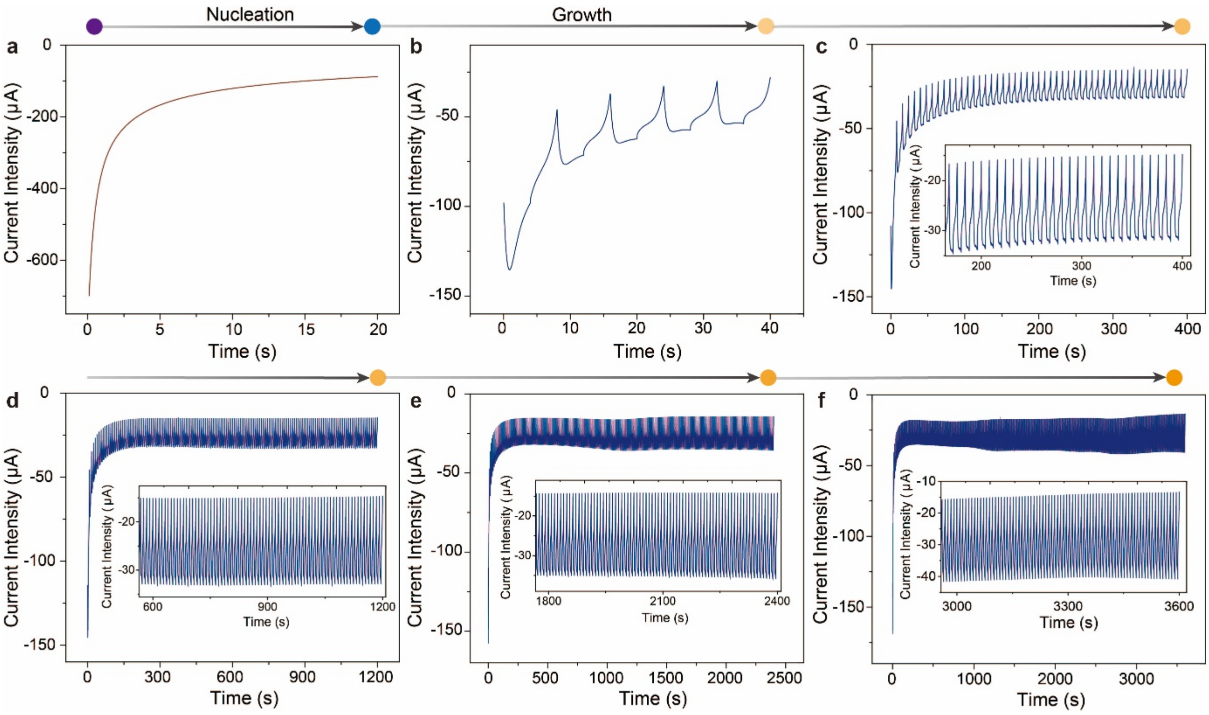

Supplementary Figure 5. The experimental current profiles for the seeding step (a) and subsequent growth step within a potential scanned range from − 0.10 to − 0.30 V (*vs.* SCE), at a scan rate of 0.05 V s^{-1}, 5 (b), 50 (c), 150 (d), 300 (e), and 450 (f) cycles. Inset showed the enlarged view of the current profile in c-f.

Supplementary Note 3：Characterization of Electrochemical Deposited Cu NPs

Electrochemical seed-mediated growth of Cu NPs was adopted to fabricated plasmonic-enhanced system for single-wavelength monitoring interfacial reactions at the electrode-electrolyte interface. Owing to the enlarged nanoparticle size, the extinction cross section increased gradually and tend to saturate after deposition 150 cycles (Figure 1, shown in main text). Therefore, the electrodes obtained by electrochemical deposition for 150 cycles were used for composition analysis and the following measurements. To provide more composition information of the electrodeposited Cu NPs, time of flight secondary ion mass spectrometry (ToF-SIMS) measurements were performed to determine that the elemental composition of the nanostructures consisted of primarily Cu (Supplementary Figure 7, a). The isotope imaging of $^{63\wedge}$Cu and $^{65\wedge}$Cu on the electrode showed consistent value with the natural isotope distribution of Cu and indicated that the copper nanoparticles were homogeneously distributed on the electrode surface without obvious aggregation and agglomeration due to the emulative nucleation processes under severe cathodic polarization (Supplementary Figure 7). The electrodeposited Cu nanoparticles were further characterized by powder X-ray diffraction (XRD) and despite the diffraction spectrum exhibited weak crystallinity, the characteristic diffraction peak assigned to Cu (111) facet was still recognized (Supplementary Figure 7, b). Supplementary Figure 7, c showed the high-resolution image of Cu NPs. The lattice fringes *ca.* 0.21 nm could be indexed to the Cu (111) plane of face-centered cubic Cu, which indicated that the major exposed planes were Cu (111) of face-centered cubic Cu in accordance with the above XRD results. In addition, high-angle annular dark-field imaging and energy-dispersive X-ray spectroscopy (EDX) analysis revealed Cu NPs possessed a nanosphere morphology with a diameter of *ca.* 160 nm which was agreed well with the AFM measurements (164 nm) (Figure 1). Due to the relative lower cathodic polarization window from – 0.10 to – 0.30 V *vs.* SCE applied for the Cu nuclei growth, it could explain the growth of the Cu NPs were mainly exposed by Cu (111) facet because of its low surface energies insufficient to facilitate the growth of high-index facets.[16]

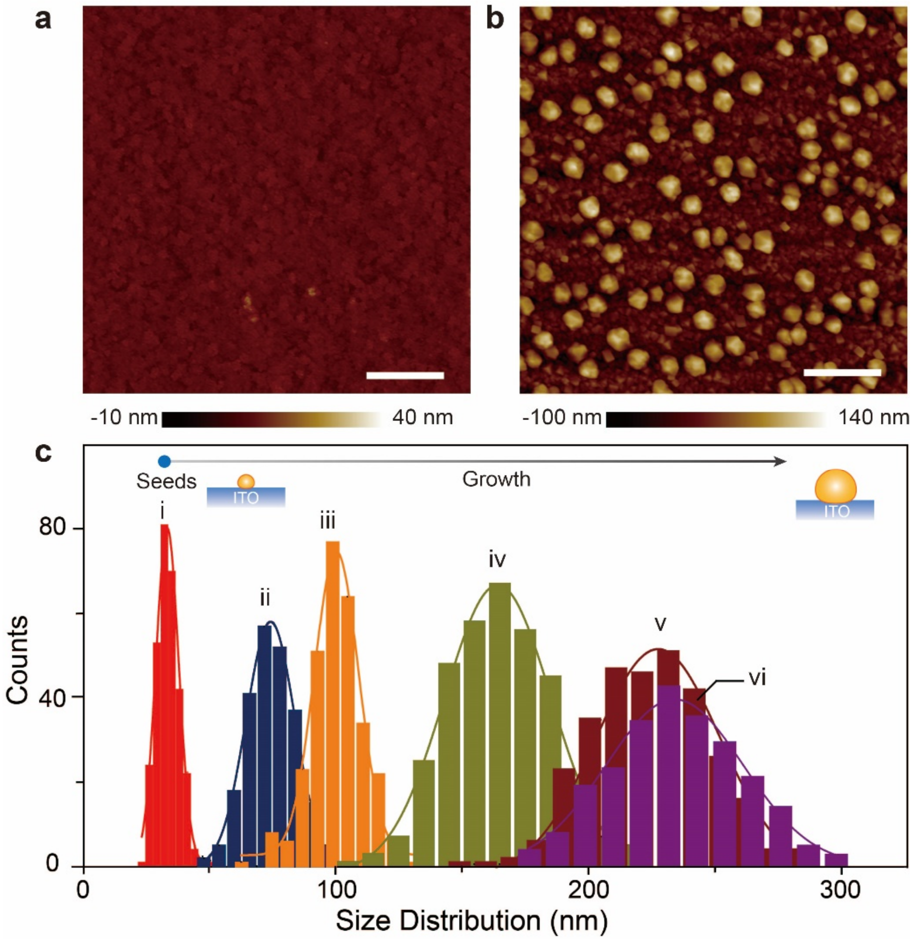

Supplementary Figure 6. The AFM images of a bare ITO electrode (a) and Cu nanoparticles (b) with 450 deposition cycles (scale bar is 1 μm). (c) The size distribution of Cu seeds (i) and Cu nanoparticles at different deposition cycles (ii 5, iii 50, iv 150, v 300, and vi 450 cycles). Inset showed schematic of Cu nanoparticle structure on the ITO electrode. Note that the generation of Gaussian size distribution was caused by the fluctuations in Cu NPs growth, including the evolution of local heterogeneous diffusion layer around Cu nuclei and concurrent Ostwald ripening-like effect.

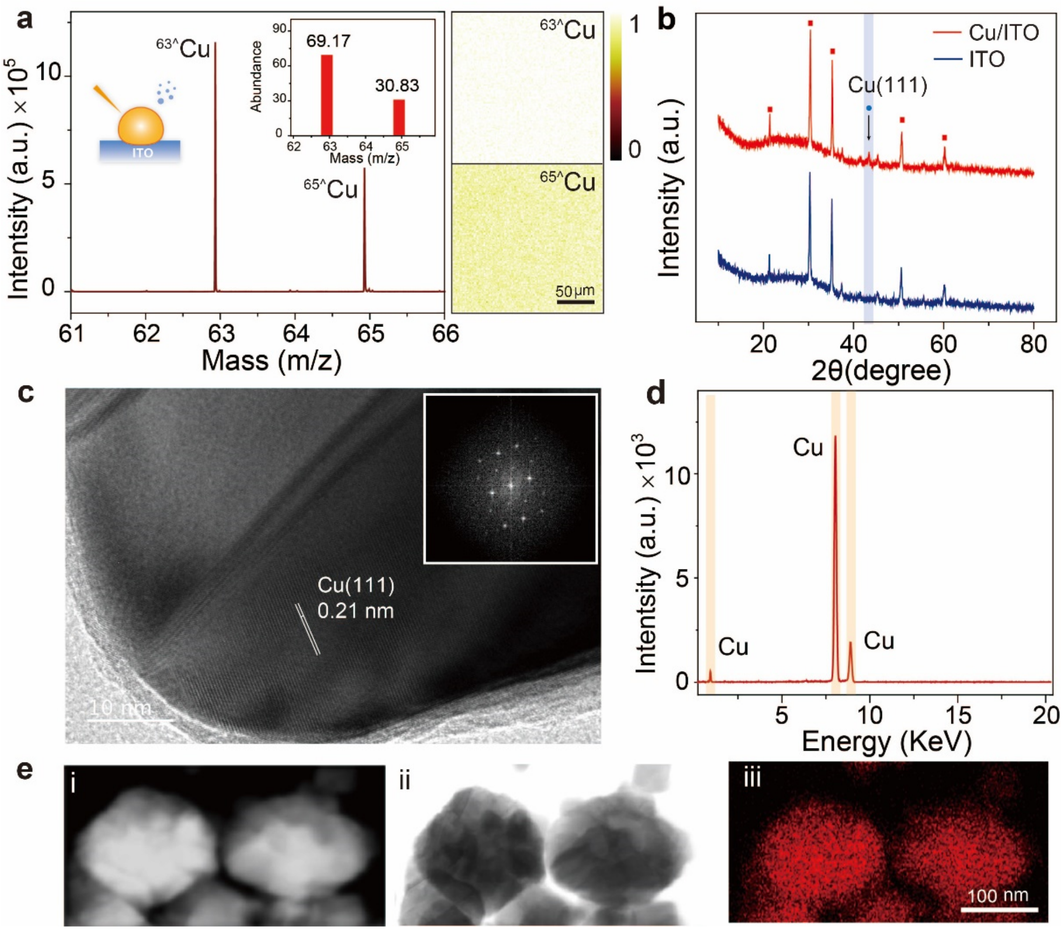

Supplementary Figure 7. (a) Negative secondary ion mass spectra of Cu species on the Cu NPs/ITO electrode. The presence of Cu in the formed nanoparticle was confirmed by the isotope peaks of Copper on the ITO electrode (^{63}^Cu and ^{65}^Cu). It was same with the natural isotope distribution of copper (Inset). The imaging of Cu isotopes (^{63}^Cu and ^{65}^Cu) were shown on the right panel. The homogenous distribution of Cu isotopes indicated the homogeneity for the electrochemical deposited Cu NPs on the ITO electrode. (b) X-ray diffraction patterns of the ensemble Cu NPs/ITO electrode (red line) and bare ITO electrode (bare line). The 2θ peak at 43.3° corresponding to the (111) planes of the Cu nanoparticle (PDF 00-004-0836) was further confirmed by the HR-TEM image. (c) HR-TEM image of the electrochemical deposited Cu nanoparticles. Inset showed the corresponding fast Fourier transform (FFT) pattern of Cu (111). HR-TEM images exhibited the interplanar distances of Cu (111) lattice planes, confirming the electrochemical deposited Cu nanoparticle were mainly composed by the Cu (111) plane. The slight surface oxides were caused by exposure to air during transport to the TEM. (d) EDX spectrum of the ensemble Cu nanoparticles. (e) High-angle annular dark-field imaging image (i), HR-TEM image (ii) and EDX mapping (iii) of electrochemical growing Cu NPs.

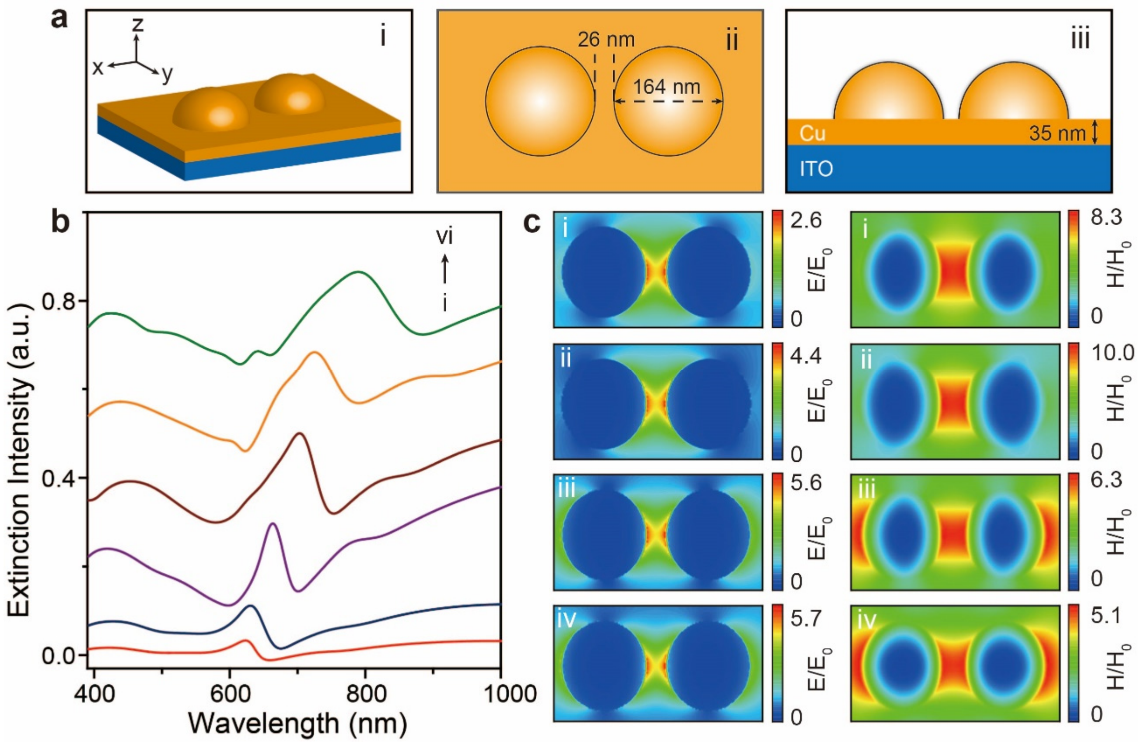

Supplementary Figure 8. (a) Schematic of proposed plasmonic-enhanced system. (i) Schematic of the plasmonic-enhanced system consisting of the Cu nanoparticles, Cu thin film, and ITO electrode. (ii) Top-view of the representative standalone plasmonic structure with geometric parameters. (iii) Cross-sectional side-view of the device. (b) Simulated extinction spectra of a standalone Cu dimer and thin film on an ITO substrate. The diameter of the nanodimer was set to 34, 74, 100, 164, 227, 234 nm from i to vi, with a 35 nm thickness and 26 nm gap in between. (c) Top-view electric (i-iv, left panel) and magnetic (i-iv, right panel) field snapshots for the near field confinement at the relative plasmonic resonance wavelengths (i: 900 nm, ii: 800 nm, iii: 500 nm, and iv: 400 nm) in Figure 1e.

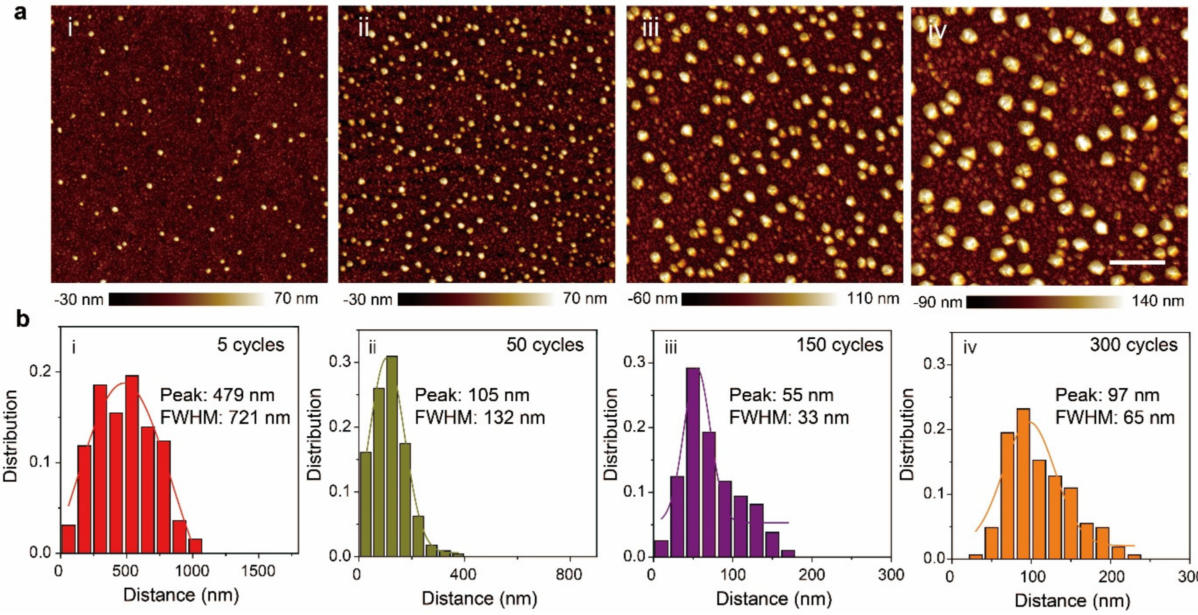

Supplementary Figure 9. (a) Representative AFM images of Cu NPs at different deposition cycles (i 5, ii 50, iii 150 and iv 300 cycles) (scale bar: 1 μm). (b) The corresponding gap distribution (peak location and full width at half maxima, FWHM) of electrodeposited Cu NPs. The minimum gap between two adjacent nanoparticles was chosen for statistical analysis.

To quantitatively describe the gap distribution between adjacent Cu nanoparticles, we provided a statistical analysis on the average particle-to-particle distances (Supplementary Figure 9). According to the definition of gap, only the two adjacent NPs with a minimum distance comparable to surrounding particles are considered as a candidate dimer for statistical analysis. From the statistical analysis we found a decrease of the gap from 479 nm to 55 nm with deposition cycles from 5 to 150 cycles accomplished with a gradual growth of particle size, and a slightly increase of gap from 55 nm to 97 nm with deposition cycles from 150 to 300 cycles caused by the Ostwald ripen-like effect.[17] In order to eliminate the contribution from random dispersed particles induced over-evaluation on the gap distance, we adopted half of the gap distance (26 nm for Cu NPs/ITO, 150 cycles) in simulation. From the simulation results (Supplementary Figure 8), similar spectral evolution trend was achieved compared to experimental measurements (Figure 1e).

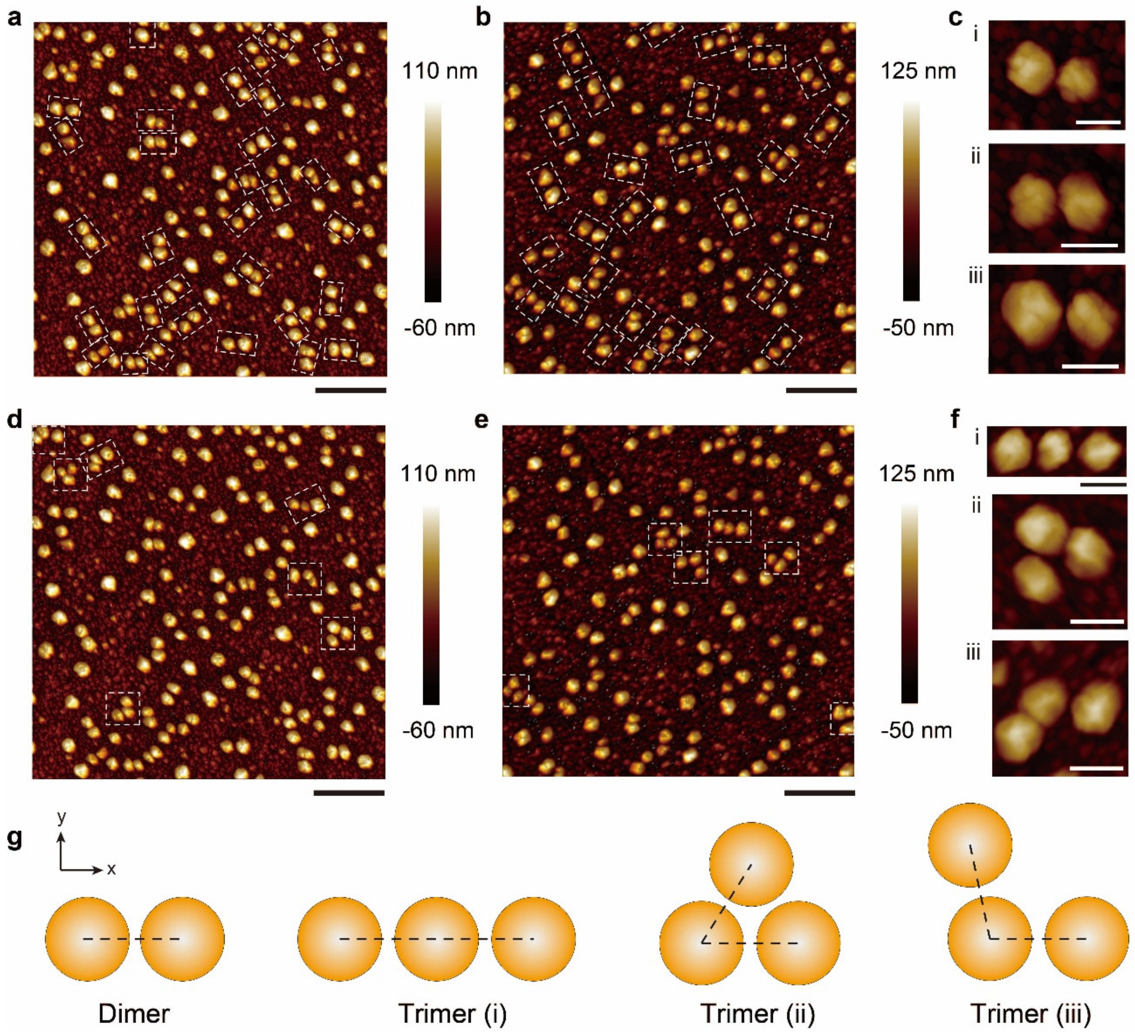

Supplementary Figure 10. (a) and (b) Representative AFM images of Cu NPs at deposition cycles (150 cycles) (scale bar: 1 μm). The isolated dimer structure was marked by white rectangle (the amount of marked isolated dimer: a 31; b 28). (c) Magnified images of representative dimer structure (i-iii) (scale bar: 200 nm). (d) and (e) Representative AFM images of Cu NPs at deposition cycles (150 cycles) (scale bar: 1 μm). The isolated trimer structure was marked by white rectangle (the amount of marked isolated trimer: d 7; e 6). (f) Magnified images of representative trimer structure (i-iii) (scale bar: 200 nm). (g) The spatial configuration of both dimer and trimer. Owing to the increase of the symmetry of trimer, more possible configurations are present in the ensemble system, which result in the extreme difficulty in distinguish their contribution to optical characteristics.

Here, a seed-mediated electrochemical deposition strategy instead of electron beam evaporation or other photolithography techniques, was used to fabricate the particles-on-film structure. Due to the intrinsic limitation of non-template electrochemical method, the formation of dispersed particles and trimers is not avoidable. The electrodeposited structures, such as dimer and timer on the film, could also attribute to the optical properties of the ensemble system. We compared the distribution and amount of isolated dimer and isolated trimer (per μm^2) on Cu NPs/ITO electrode (150 cycles). Statistical analysis indicated that the isolated dimer was

more widely distributed, and its cover density (per μm^2) was *ca.* 4.5 times higher than that of isolated trimer (Supplementary Figure 10, Dimer (31+28) / Trimer (7+6) ≈ 4.5). Considering the higher cover density and limited configurations of isolated dimer, we chose the isolated dimer as typical individual unit for simulation. As demonstrated in the simulation results (Supplementary Figure 8), we found a similar evolution trend as a function of particle size, which was in good agreement with experimental measurements (Figure 1e, main text). Thus, we tentatively concluded that the proposed model of isolated dimer-on-conductive substrate structure could represent key spectral feature of the ensemble system.

Nonetheless, we admit a more careful considering of the isolated trimer or more complicated chain structure could move the simulation results even closer and more compatible to real optical performance of the ensemble system. Considering the possible multi-configurations of the trimer (Supplementary Figure 10) or chain structure, a more detailed distinguish their contribution to the ensemble system is far beyond the aim of present study, which needs more complicated modeling and combination of single particle spectroscopy techniques to correlate both simulation and experimental results.

Supplementary Note 4：Oxygen Evolution on the Cu NPs Electrodes

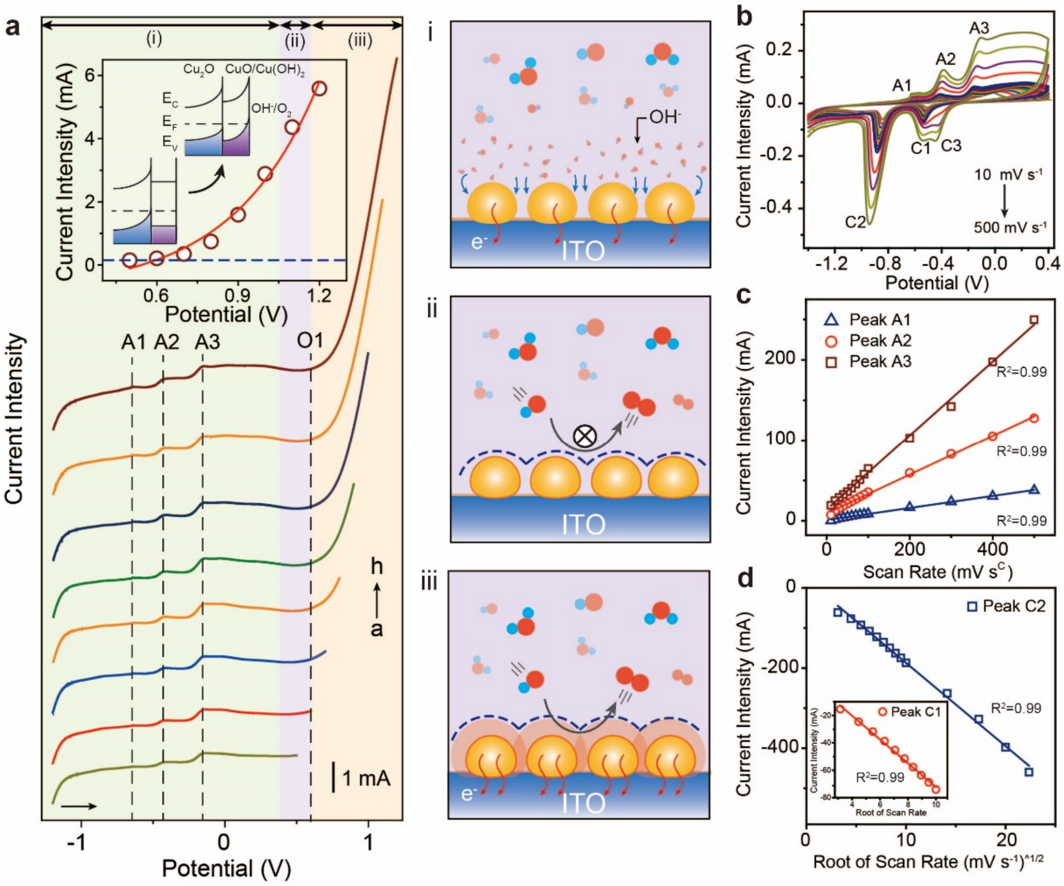

Supplementary Figure 11. Oxygen evolution reaction on Cu NPs electrode. (a) The linear scan curves for a Cu NPs/ITO electrode in 0.10 M KOH solution with various termination potential (a-h: 0.50, 0.60, 0.70, 0.80, 0.90, 1.00, 1.10 and 1.20 V), at a scan rate of 0.01 V s^{-1} (the graphs were vertically shifted for ease of comparison). Inset: the band gap transition in the transpassive region. The schematic illustrations of various interfacial processes during oxygen evolution reaction (i-iii). (b) Scan rate dependence of CV curves for a Cu NPs/ITO electrode in a solution of 0.10 M KOH. Scan rate: 0.01, 0.02, 0.03, 0.04, 0.05, 0.06, 0.07, 0.08, 0.09, 0.10, 0.20, 0.30, 0.40, and 0.50 V s^{-1}. (c) The dependence of anodic peak current at peak A1, A2, and A3 on scan rate. (d) The dependence of cathodic peak current at peak C1 and C2 on the square root of the scan rate.

Linear potential scan measurements were used to track dynamic interfacial processes at the EEIs (Supplementary Figure 11). The dynamic interfacial process exhibited three characteristic regions with increasing potential: (1) At the interface active region (< 0.30 V *vs.* Pt unless otherwise stated, all the potential referenced to a Pt quasi-reference electrode), the process of OH^- adsorption took place, assigning to the peak located at − 0.63 V (peak A1). With the increasing of potential to greater than − 0.39 V, the adsorbed phase on the copper surface eventually reconstructed, resulting in the formation of a Cu_2O layer, which was indicated by the presence of peak A2.[18] With further enhancement in anodic polarization, the arising of broad peak A3 was attributed to the accompaniment of both electrooxidation of Cu(I) to Cu(II) and the formation of CuO and $Cu(OH)_2$ (Region i, a detailed kinetic analysis shown in Supplementary Figure 11); (2) At the interfacial passive region (0.30 V ~ 0.60 V), the formation of a thicker and complex Cu oxides and hydroxides passive film restrained the interfacial oxidation, and no obvious catalytic activity towards oxygen evolution was observed (Region ii); (3) At the interfacial transpassive region (> 0.60 V), the gradually enhanced dehydration process accompanied by a structural transformation from an amorphous to a crystalline structure, providing an electron-conductive pathway for oxygen evolution (Region iii). The crossing of the $CuO/Cu(OH)_2$ layer valence band and the Fermi level caused an enrichment of positive holes in this transpassive region, that significantly improved the oxygen evolution efficiency (Supplementary Figure 11, a inset). The proposed interfacial reaction mechanisms on Cu NPs electrode in alkaline solution were expressed as:

$$\mathrm{Cu} + OH^- \rightarrow Cu(OH)_{ad} + e^- \tag{4-1}$$

$$2Cu + 2OH^- \rightarrow \mathrm{Cu_2O} + \mathrm{H_2}O + 2e^- \tag{4-2}$$

$$\mathrm{Cu_2O} + \mathrm{H_2}O + 2OH^- \rightarrow 2Cu(OH)_2 + 2e^- \tag{4-3}$$

$$\mathrm{Cu_2O} + 2OH^- \rightarrow 2CuO + \mathrm{H_2}O + 2e^- \tag{4-4}$$

$$Cu(OH)_2 \rightarrow CuO + \mathrm{H_2}O \tag{4-5}$$

The influence of the sweep rate on both anodic and cathodic polarization was shown in Supplementary Figure 11, b. The cathodic peaks C1 was assigned to the electrochemical reduction of CuO to Cu_2O. The other peak C2 was caused by the simultaneous reduction of both Cu_2O and CuO to Cu.[19] During kinetic analysis, a linear relationship between anodic peak currents and the scan rate indicated a surface reaction controlled kinetic in anodic processes

(Supplementary Figure 11, b, Peak A1, A2 and A3). Meanwhile, the cathodic processes (Peak C1 and C2) exhibited a linear function as the square root of the scan rates, indicating a diffusion-controlled mass transport in these processes (Supplementary Figure 11, c). Interestingly, a gradually emerging cathodic peak located at − 0.45 V was found at relative high scan rate (> 0.20 V s^{-1}) which was assigned to the reduction from $Cu(OH)_2$ to Cu_2O. Taken together with kinetic analysis of interfacial reactive species, the linear scan voltammetry enabled to reasonably elucidate their compositions and distinguish controlled manner of interfacial reactions under polarization.

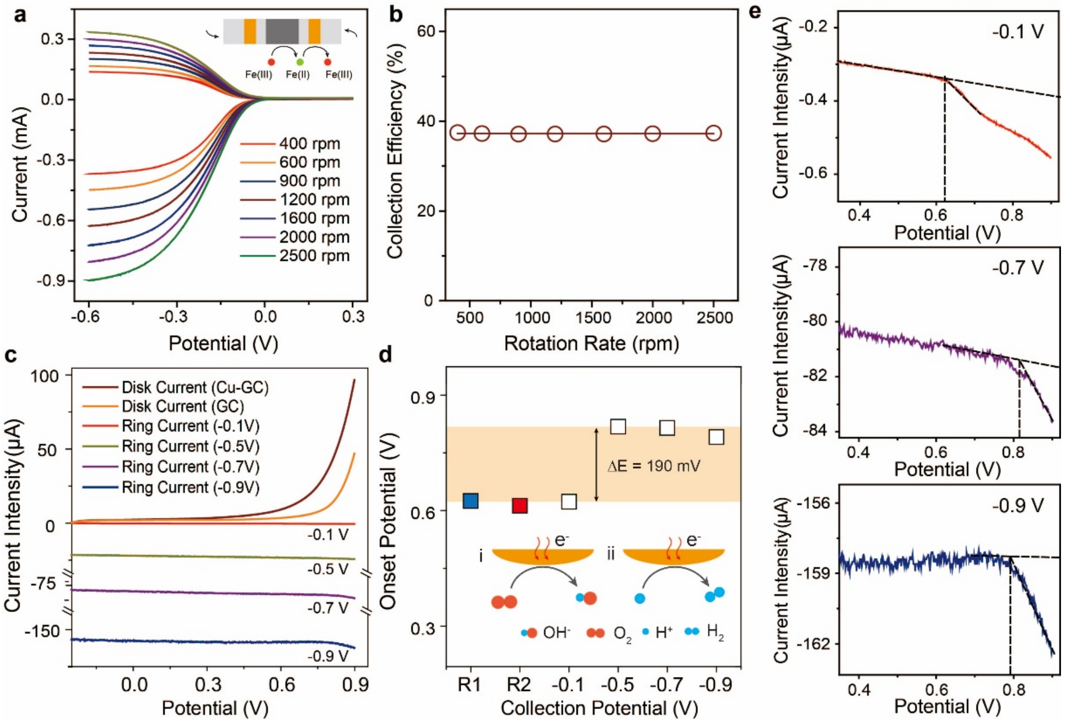

Supplementary Figure 12. The evaluation of the apparent ring collection efficiency for a glass carbon-disc and Pt-ring RRDE. (a) The CVs of the glass carbon-disc in 0.10 M KOH solution containing 5 mM $K_3[Fe(CN)_6]$ at 0.01 V s^{-1} under different rotation rates (400, 600, 900, 1200, 1600, 2000 and 2500 rpm). Inset showed schematic of the estimation for the ring collection efficiency of a glass carbon-disc and Pt-ring RRDE. (b) The corresponding disk and ring current ratio (the apparent ring collection efficiency = I_r/I_d) at ring potential 0.30 V (*vs* Pt). (c) The linear scan curves for OER on a Cu NPs/GC disc electrode and the collection current curves at various collection potential on a Pt ring electrode. (d) The comparison of onset potential for RRDE experiments at different collection potentials and conventional linear scans for a Cu NPs/ITO (R1, blue square) and a Cu NPs/GC (R2, red square) electrode. Inset: the competing reaction of proton reduction during oxygen reduction. (e) The representative onset potential for collection of electrochemical generated oxygen on the Pt collection electrode at − 0.10, − 0.70 and − 0.90 V.

In order to estimate the collection efficiency of the RRDE, the reversible single electron transfer reaction of ferrocyanide/ferricyanide ($Fe(CN)_6^{3-}/Fe(CN)_6^{4-}$) was used as the model reaction. With the potential of the disc working electrode scanning toward to the cathodic polarized direction, the increased cathodic current was assigned to the reduction of the ferricyanide to ferrocyanide at the disc working electrode (generator). The oxidation current for products oxidation was instantaneous collected on the ring electrode (collector).

$$Fe(CN)_6^{3-} + e^- \rightarrow Fe(CN)_6^{4-} \text{ (Generator)}$$

$$Fe(CN)_6^{4-} \rightarrow Fe(CN)_6^{3-} + e^- \text{ (Collector)}$$

Hence the empirical collection efficiency (N_e) was estimated based on the limiting current ratio of the ring and disc. Both the cathodic and anodic limiting currents were proportional to the

square root of the scan rate, the evident increase of both generator and collector currents were observed (Supplementary Figure 12, a). Owing to the independence of empirical collection efficiency on the rotation rate, the estimated empirical collection efficiency *ca.* 37 % was nearly constant under different rotation rate (Supplementary Figure 12, b).

We further performed rotating-ring disc electrode (RRDE) measurements to collect oxygen at various polarization potentials to explore more insights into OER. We found that at collection potential − 0.10 V *vs.* Pt, the collected current on Pt ring electrode showed negligible cathodic current only noticeable increasing beyond 0.60 V which was assigned to the oxygen reduction and indicated an onset potential at 0.60 V of oxygen evolution at the disk electrode. It was consistent with the value extracted from the linear potential scan results (Supplementary Figure 11, a). As decreasing the collection potential, the coupled reactions *via* co-electrolysis of protons on the Pt ring electrode restrained OER performance and resulted in a negative shift of *ca.* 0.19 V in the onset potential of OER (Supplementary Figure 12). We emphasized that the deterioration in the onset potential for oxygen collection suggested a significant exacerbated competing reaction kinetic between the oxygen and proton reduction. Furthermore, the polarization current increased *ca.* 69.5 times from − 0.10 to − 0.90 V indicated the evident enhancement in the hydrogen evolution reaction (HER) rate. Such an increased cathodic polarization on this scale resulted in a significant rise in interfacial reaction rates of competing hydrogen evolution reaction assuming the reaction was kinetically controlled.[20-23] Similar results were observed in co-electrolysis of protons with the addition of O_2, which provided direct evidence for the presence of oxygen in RRDE experiments.[24] Such significant suppression for oxygen collection by competing co-electrolysis reaction hampered the accurate identification of oxygen at the EEIs.

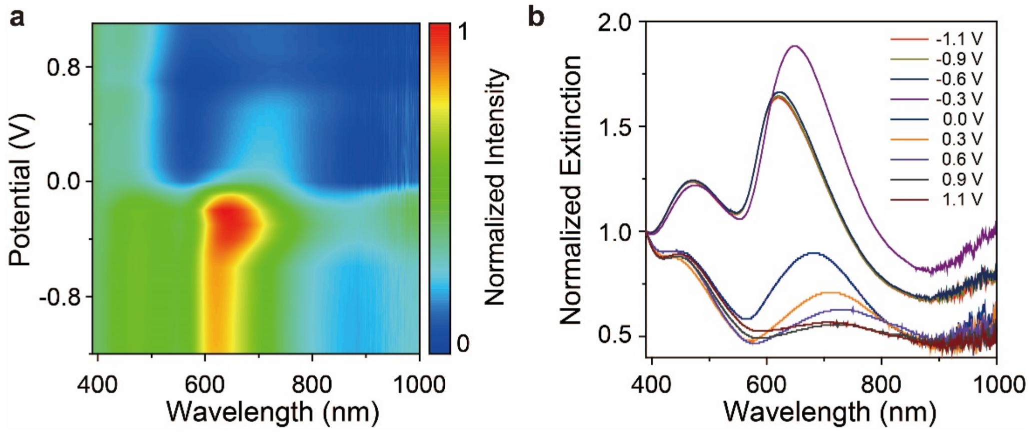

Supplementary Figure 13. (a) The normalized dynamic extinction intensity (contour plots) of a Cu NPs/ITO electrode under steady-state anodic polarization from − 1.20 V to +1.10 V. (b) The representative normalized extinction spectra at various polarized potential.

Here we aimed to use electrochemical steady-state extinction spectroscopy to monitor OER at electrode surface (Figure 2b and Supplementary Figure 13). With the enhancement of anodic polarization, the spectral intensity of Cu NPs/ITO decreased gradually. The dynamic spectral changes indicated that the Cu oxide and hydroxides intermediates were weakly bound and readily dissolved during long time anodic polarization, which hampered interfacial processes dissection. Thereby, it prompts us to develop an alternative single-wavelength plasmonic monitoring strategy to precisely identify the diffusion behavior of dissolved oxygen to avoid the persistent oxidation of the electrode surface under electrochemical steady-state condition.

Supplementary Note 5: Principle of the Single-Wavelength Monitoring Strategy

The plasmonic properties of metal nanoparticles can be described by solving the Maxwell equation using classical electrodynamic Mie theory.[25-26] According to Mie theory, the analytical solution to the scattered fields generated by a plane wave incident on a homogeneous plasmonic nanoparticle leaded to the extinction, scattering and absorption cross-section[27-28]: the general expressions for the scattering and extinction cross sections from the Mie theory can be expressed as

$$\sigma_{ext} = \frac{2\pi}{|k|^2}\sum_{L=1}^{\infty}(2L+1)\,Re[a_n + b_n] \tag{5-1}$$

where k is the incoming wavevector and L are integers corresponding to the diploe, quadrupole and hexapole contributions etc. Here, the a_L and b_L composed of Riccati-Bessel functions are given by:

$$a_L = \frac{m\Psi_L(mx)\Psi_L'(x) - \Psi_L'(mx)\Psi_L(x)}{m\Psi_L(mx)\zeta_L'(x) - \Psi_L'(mx)\zeta_L(x)} \tag{5-2}$$

$$b_L = \frac{\Psi_L(mx)\Psi_L'(x) - m\Psi_L'(mx)\Psi_L(x)}{\Psi_L(mx)\zeta_L'(x) - m\Psi_L'(mx)\zeta_L(x)} \tag{5-3}$$

where $m=n_n/n_m$, n_n and n_m is the refractive index of the nanoparticle and surrounding medium, respectively. And $x = Rk_m$, R is the radius of the nanoparticle, k_m is the wavenumber of incident light. When the size of the plasmonic nanoparticles is relative smaller than the wavelength of the incident light, taking into account the contribution from dipole term, the extinction cross-section of the plasmonic particle can be given by:

$$\sigma_{ext}(\omega, R) = 18\pi\frac{\omega V\varepsilon_m^{3/2}}{c}\frac{\varepsilon_2(\omega,R)}{[\varepsilon_1(\omega,R)+2\varepsilon_m]^2+\varepsilon_2(\omega,R)^2} \tag{5-4}$$

where c is the speed of the light and R is the radius of the nanoparticle, V is the volume of the nanoparticle, ω is the frequency of the incident light and ε_m is the dielectric constant of the surrounding medium, ε is the dielectric constant of the nanoparticle, $\varepsilon = \varepsilon_1 + i\varepsilon_2$. ε_1 and $i\varepsilon_2$ are real and complex parts of the material dielectric constant, respectively.[27] Based on this equation, the optical properties of the plasmonic nanoparticles can be engineered by tuning their size and dielectric properties of the surrounding and material of the nanoparticle.[29] The extinction cross-section will increase when the medium's dielectric function decrease. The dependence of the LSPR extinction cross-section on the surrounding dielectric environment enables the ultrasensitive detection of the minor changes in surrounding medium. Note that the plasmonic

extinction cross-section is proportion to a negative half power of dielectric function of surrounding medium. Conventionally, the plasmonic sensing application is to detect changes in the bulk refractive index of surrounding environment by monitoring the LSPR peak wavelength shifts through spectroscopic extinction measurements (eq. 5-5 and 5-6).

$$\omega_{max} = \frac{\omega_p}{\sqrt{2\varepsilon_m + 1}} \tag{5-5}$$

$$\lambda_{max} = \lambda_p \sqrt{2n_m^2 + 1} \tag{5-6}$$

where ω_{max} is the LSPR peak frequency and ω_{p} is the plasma frequency of the bulk metal. The λ_{max} and λ_{p} corresponding to the wavelength of LSPR peak and the plasma frequency of the bulk metal. Here, the $\lambda = 2\pi c/\omega$ and the relationship between dielectric constant and index of refraction $\varepsilon_{\text{m}} = n^2$. Hence, the LSPR peak wavelength shift is approximately linear with changes in refractive index of the surrounding medium over small ranges of *n*.[28] For collection full extinction spectra, the monochromator and grating as well as multichannel photodetector are essential to identify the plasmonic peak shifts. In order to facilitate the in-situ spectroscopic monitoring interfacial electrochemical reactions, the single-wavelength extinction monitoring strategy was developed with superior sensitivity for electrochemical reactions on the EEIs and eliminated the necessity for intricate optical apparatuses.

Figure 3c showed a schematic relationship between extinction (*I*), potential (*E*) and wavelength (λ) (I-λ–E plot) for a plasmonic nanoparticle, where the extinction axis was vertical to the sheet. During the dynamic potential scan, the interfacial processes at the EEIs induced the fluctuation of the refractive index nearby the electrode surface, which leaded to a systematic transition from original state (S1, I_1–λ_1–E_1) to reaction condition (S2, I_2–λ_2–E_2). With known the relationship between the refractive index and the extinction peak intensity at a certain wavelength, it enabled the optical probing interfacial electrochemical reactions on the electrode surface which constituted by plasmonic components. As the interfacial reaction proceeding, the fluctuation of the confined products concentration resulted in a remarkable change of the refractive index around the plasmonic elements, which contributed significantly to the dynamics in extinction spectra. For instance, the electrochemically generated gas phase species on the plasmonic elements excluded the surrounding electrolyte and decreased the refractive index. It resulted in a prominent enhancement of its extinction intensity at a constant λ. Of note,

the plasmonic elements in our system not only served as the electrochemical catalytic active sites for electrochemical reaction, but also as the optical module for signal transduction in which electrochemical reaction information was convert to remarkable optical signal. Therefore, based on the fluctuation of the extinction intensity at a constant λ during dynamic potential scan, it enabled ultrasensitive feedback of the interfacial reaction information on the EEIs.

Supplementary Note 6：In-situ Extinction Spectroelectrochemical Analysis

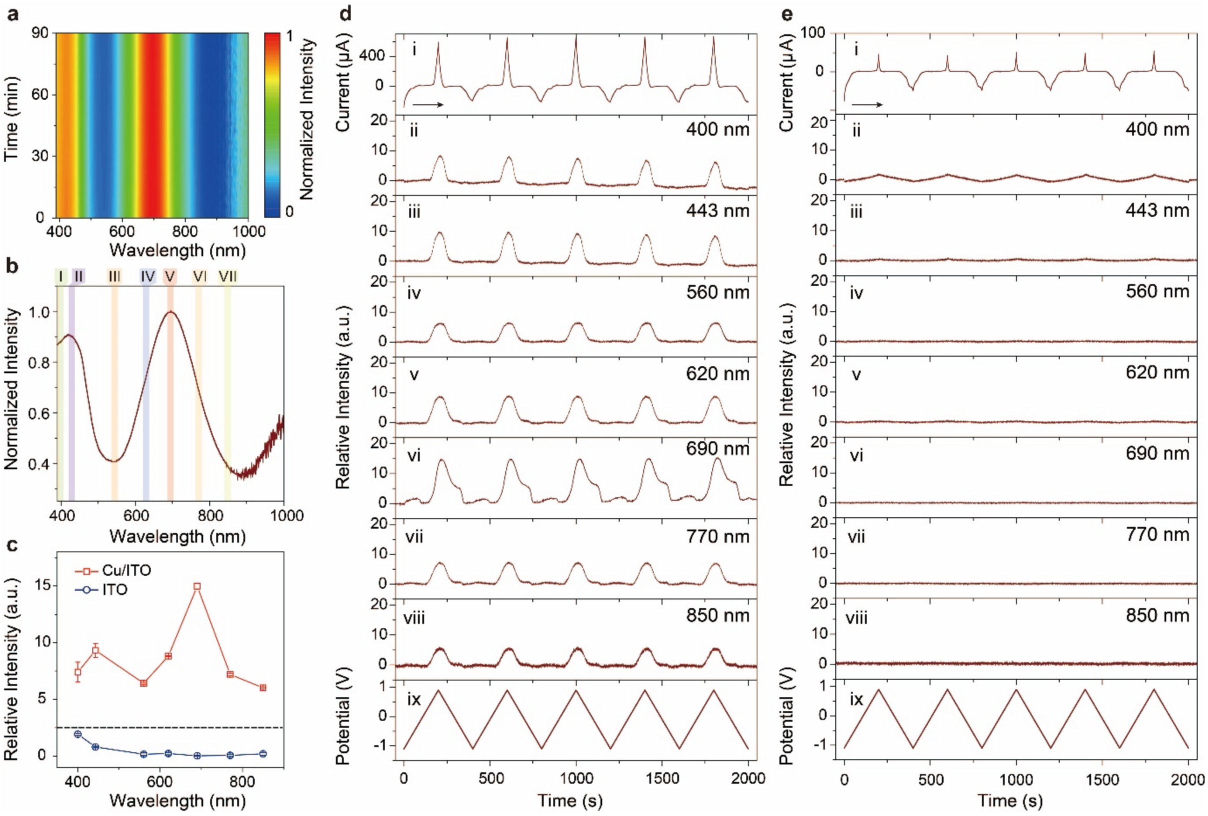

Supplementary Figure 14. (a) The stability of the extinction spectrum of a representative Cu NPs/ITO electrode at open circuit potential during 90 min. (b) The locations of different wavelength (I 400, II 443, III 560, IV 620, V 690, VI 770 and VII 850 nm) adopted for single wavelength monitoring. (c) The comparison of the maximum spectral intensities at various monitoring wavelength (I-VII, 400 nm-850 nm). The consecutive electrochemical current (i) of a Cu NPs/ITO electrode (d) and a bare ITO electrode (e) during OER and synchronous extinction signal (ii-viii, 400 nm-850 nm) under dynamic potential scan (ix) from – 1.10 to 0.90 V at a scan rate 0.01 V s^{-1}.

We compared the different temporal extinction trajectories at various wavelength during consecutive potential scan (Supplementary Figure 14). The electrodeposited Cu NPs electrode exhibited no obvious spectral fluctuation at open circuit potential in 90 min (Supplementary Figure 14 a), suggesting excellent stability. Different wavelengths (400, 443, 560, 620, 690, 770 and 850 nm) were chosen for comparison the sensing performance of OER (Supplementary Figure 14 b). When wavelength located at the CTP mode (690 nm) was adopted for monitoring, its spectral response exhibited distinct enhancement in intensity compared with others (Supplementary Figure 14 c-e). For instance, the intensity increased *ca.* 1.60 and 2.33 times at 690 nm (CTP) compared to that of 443 nm and 560 nm, respectively. Therefore, tracking the electrochemical interfacial reaction at 690 nm (CTP) enables explicitly identifying

characteristic spectral regions for revealing the OER mechanism. The sensitive interfacial reaction sensing response at the CTP mode is attributed to its intrinsic vulnerability to dynamic environment refractive index.[30]

Moreover, control experiments on bare ITO electrode monitored at 400 nm and 443 nm, revealed weak periodic fluctuation of background signal, attributing to mass transfer of interfacial species (K^+, OH^-), while no obvious fluctuation of background signal was observed at 690 nm. The importance of CTP mode is clearly highlighted by both high sensitivities to interfacial reaction and negligible background signals.

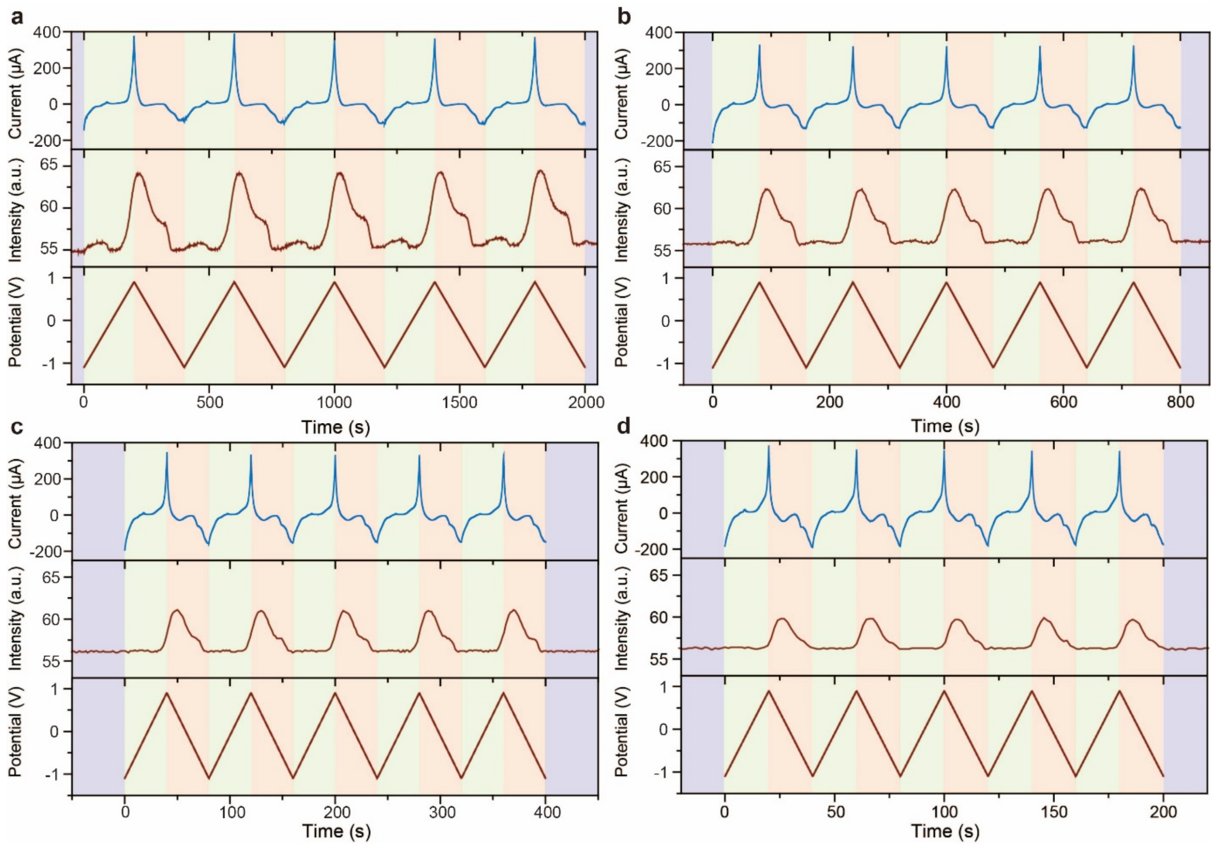

Supplementary Figure 15. Consecutive CV curves (top panel) and in-situ extinction voltammetry (middle panel) for a Cu NPs/ITO electrode in 0.10 KOH solution for oxygen evolution reaction in potential range from − 1.10 to 0.90 V *vs* Pt, at various scan rates 0.01, 0.025, 0.05 and 0.10 V s^{-1}. The applied triangle wave type potential *versus* time during scan shown in bottom panel.

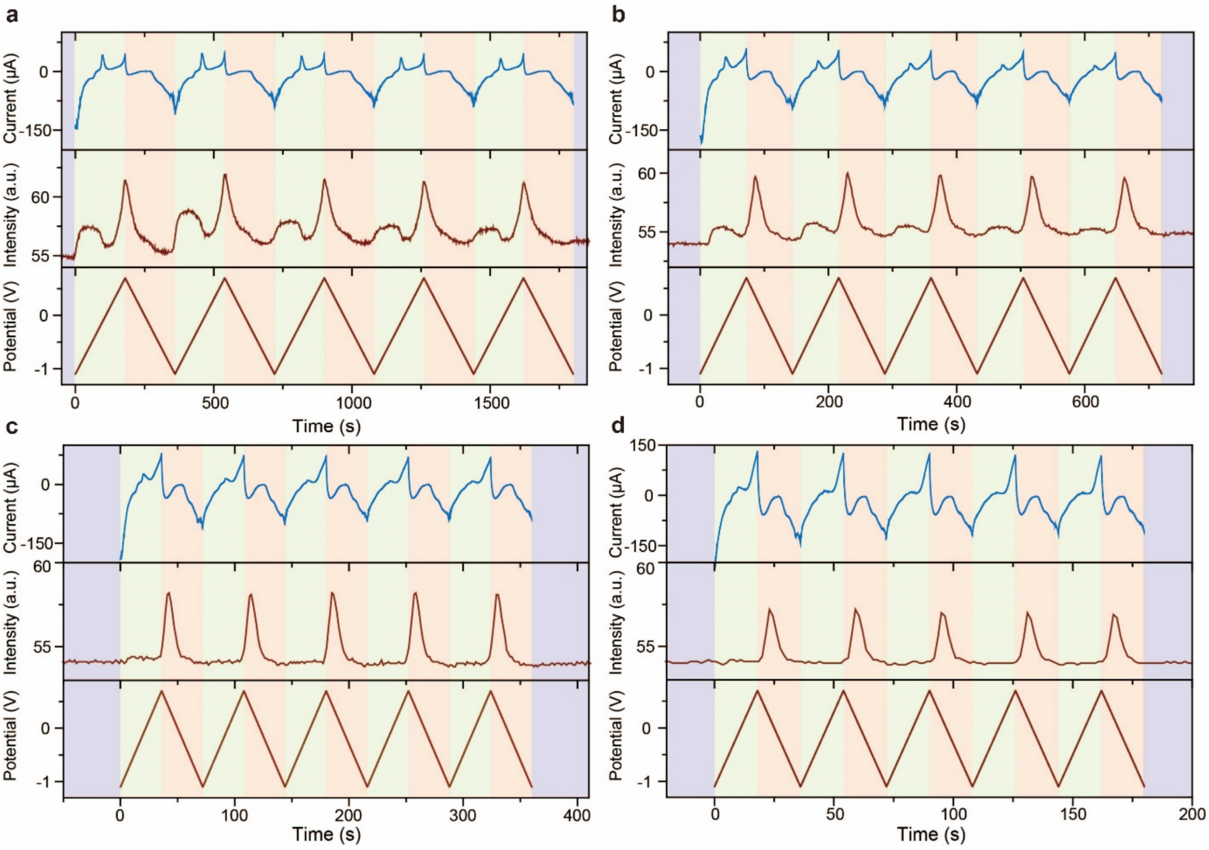

Supplementary Figure 16. Consecutive CV curves (top panel) and in-situ extinction voltammetry (middle panel) for a Cu NPs/ITO electrode in 0.10 KOH solution for oxygen evolution reaction in potential range from − 1.10 to 0.70 V *vs* Pt, at various scan rates 0.01, 0.025, 0.05 and 0.10 V s^{-1}. The applied triangle wave type potential *versus* time during scan shown in bottom panel.

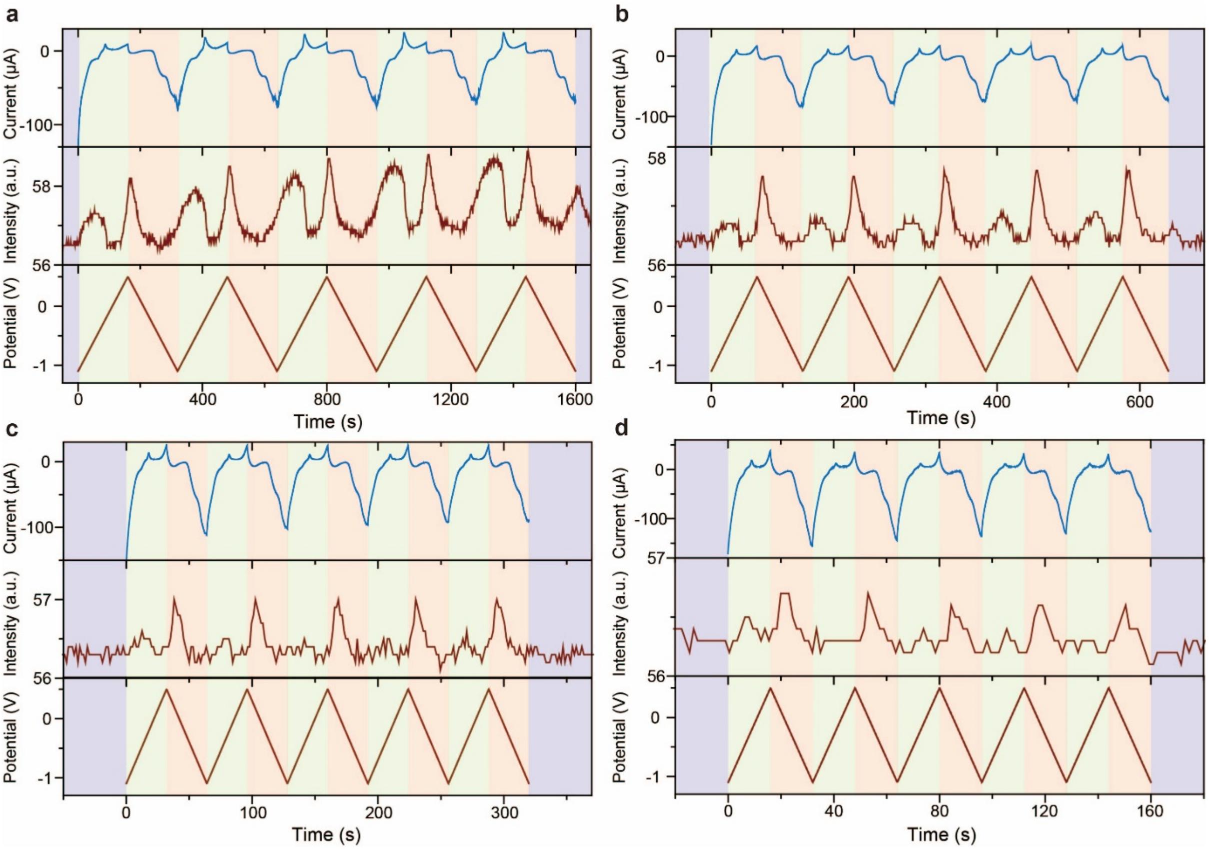

Supplementary Figure 17. Consecutive CV curves (top panel) and in-situ extinction voltammetry (middle panel) for a Cu NPs/ITO electrode in 0.10 KOH solution for oxygen evolution reaction in potential range from − 1.10 to 0.50 V *vs* Pt, at various scan rates 0.01, 0.025, 0.05 and 0.10 V s^{-1}. The applied triangle wave type potential *versus* time during scan shown in bottom panel.

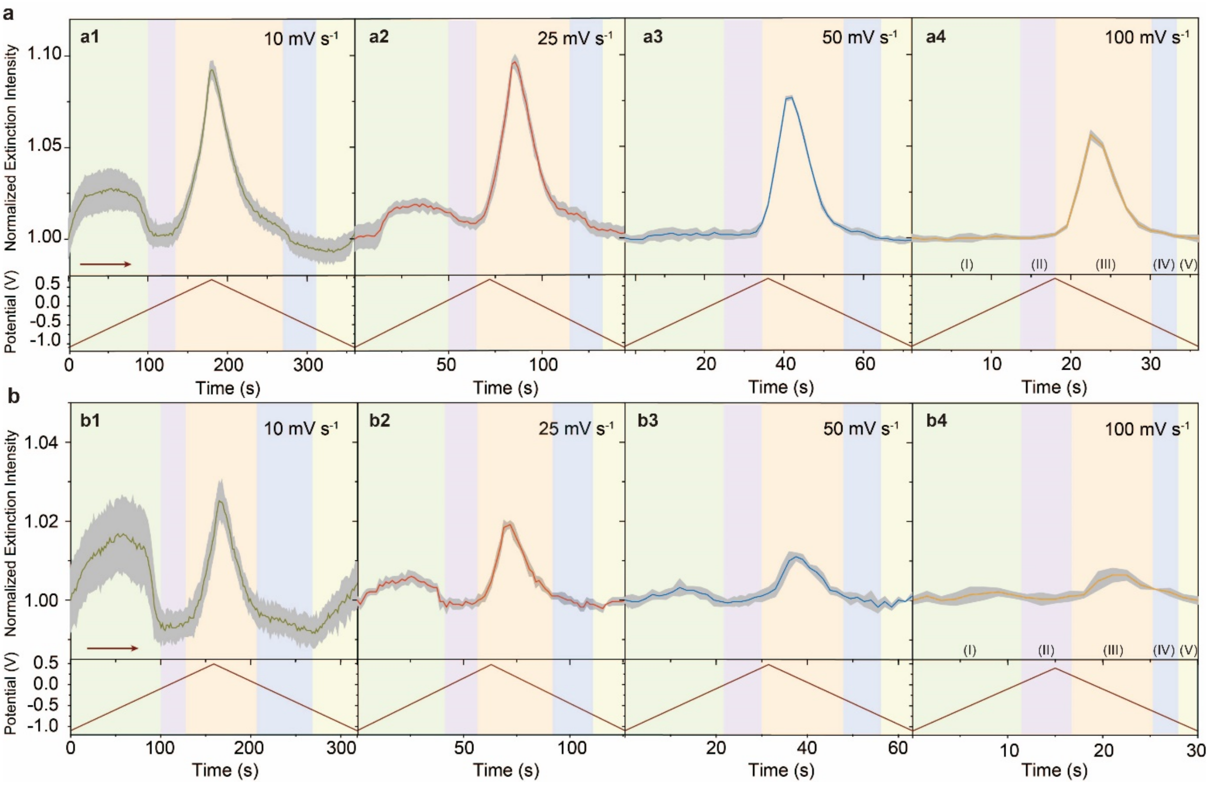

Supplementary Figure 18. (a) In-situ extinction profiles for a Cu NP/ITO electrode in 0.10 KOH solution for oxygen evolution reaction in potential range from − 1.10 to 0.70 V *vs* Pt, at various scan rates 0.01 (a1), 0.025 (a2), 0.05 (a3) and 0.10 (a4) V s^{-1}. (b) In-situ extinction profiles for a Cu NP/ITO electrode in 0.10 KOH solution for oxygen evolution reaction in potential range from − 1.10 to 0.50 V *vs* Pt, at various scan rates 0.01 (b1), 0.025 (b2), 0.05 (b3) and 0.10 (b4) V s^{-1}. The dark region represented the standard error during consecutive five potential scan.

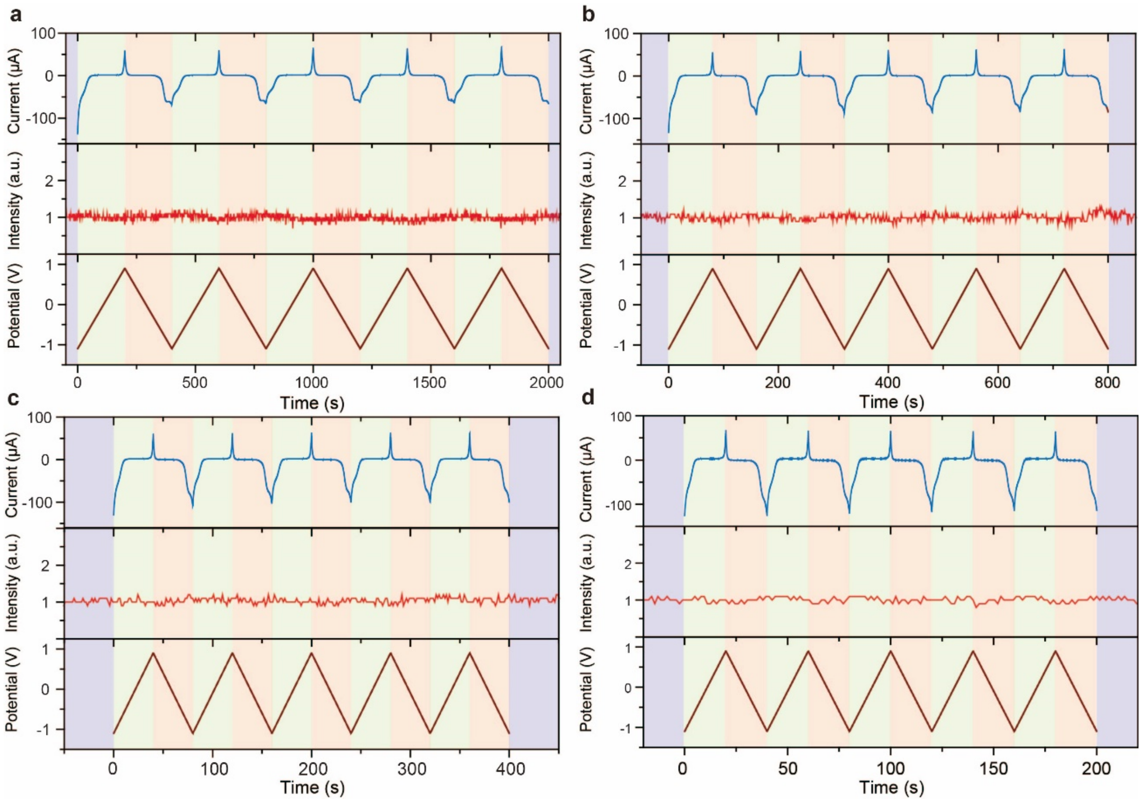

Supplementary Figure 19. Consecutive CV curves (top panel) and in-situ extinction voltammetry (middle panel) for a bare ITO electrode in 0.10 KOH solution for oxygen evolution reaction in potential range from − 1.10 to 0.90 V *vs* Pt, at various scan rates 0.01, 0.025, 0.05 and 0.10 V s^{-1}. The applied triangle wave type potential *versus* time during scan shown in bottom panel.

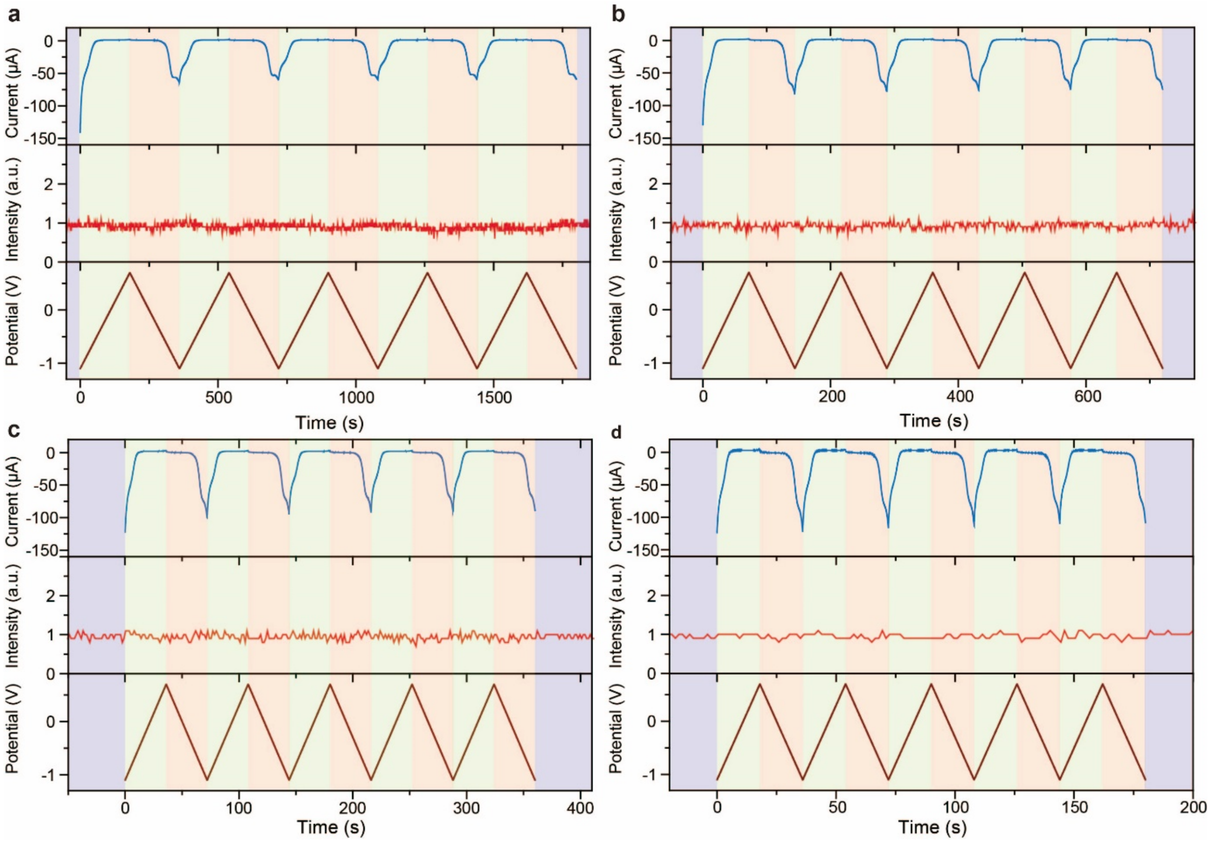

Supplementary Figure 20. Consecutive CV curves (top panel) and in-situ extinction voltammetry (middle panel) for a bare ITO electrode in 0.10 KOH solution for oxygen evolution reaction in potential range from − 1.10 to 0.70 V *vs* Pt, at various scan rates 0.01, 0.025, 0.05 and 0.10 V s^{-1}. The applied triangle wave type potential *versus* time during scan shown in bottom panel.

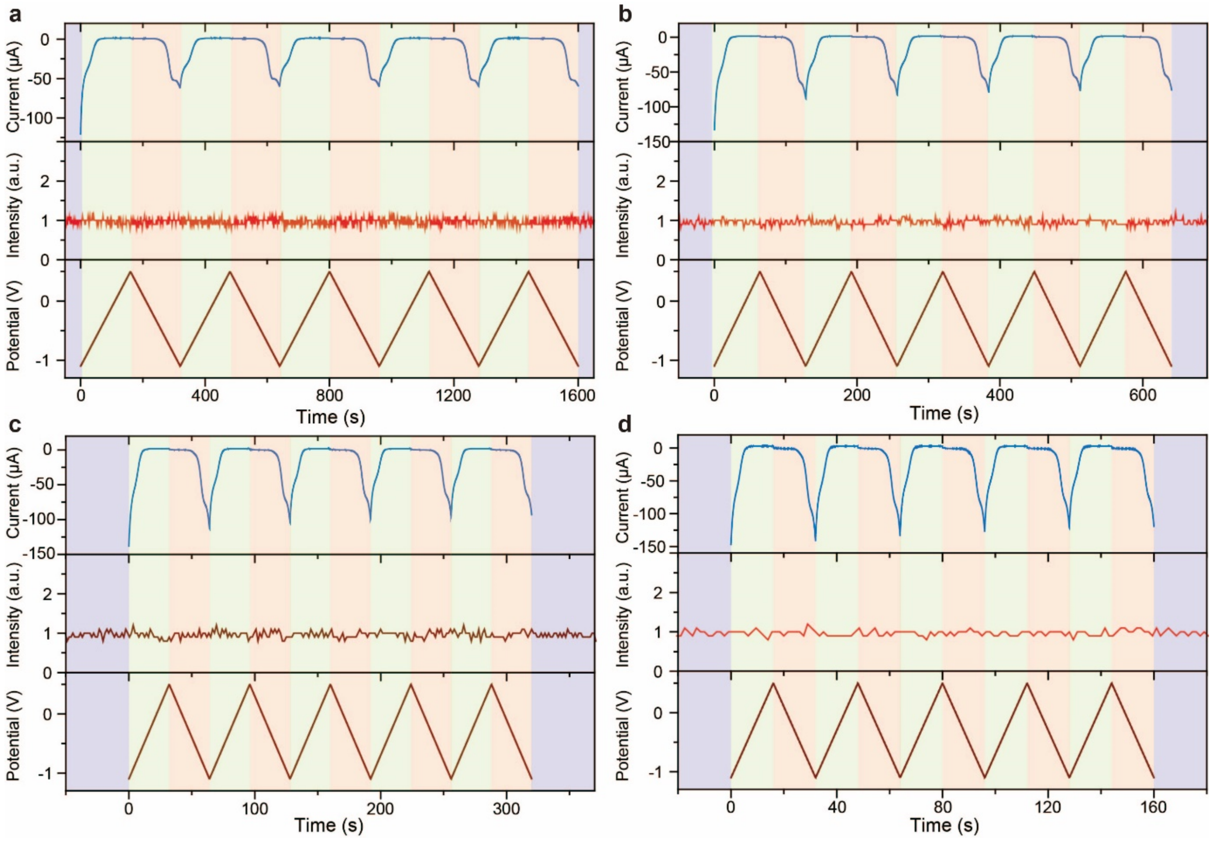

Supplementary Figure 21. Consecutive CV curves (top panel) and in-situ extinction voltammetry (middle panel) for a bare ITO electrode in 0.10 KOH solution for oxygen evolution reaction in potential range from − 1.10 to 0.50 V *vs* Pt, at various scan rates 0.01, 0.025, 0.05 and 0.10 V s^{-1}. The applied triangle wave type potential *versus* time during scan shown in bottom panel.

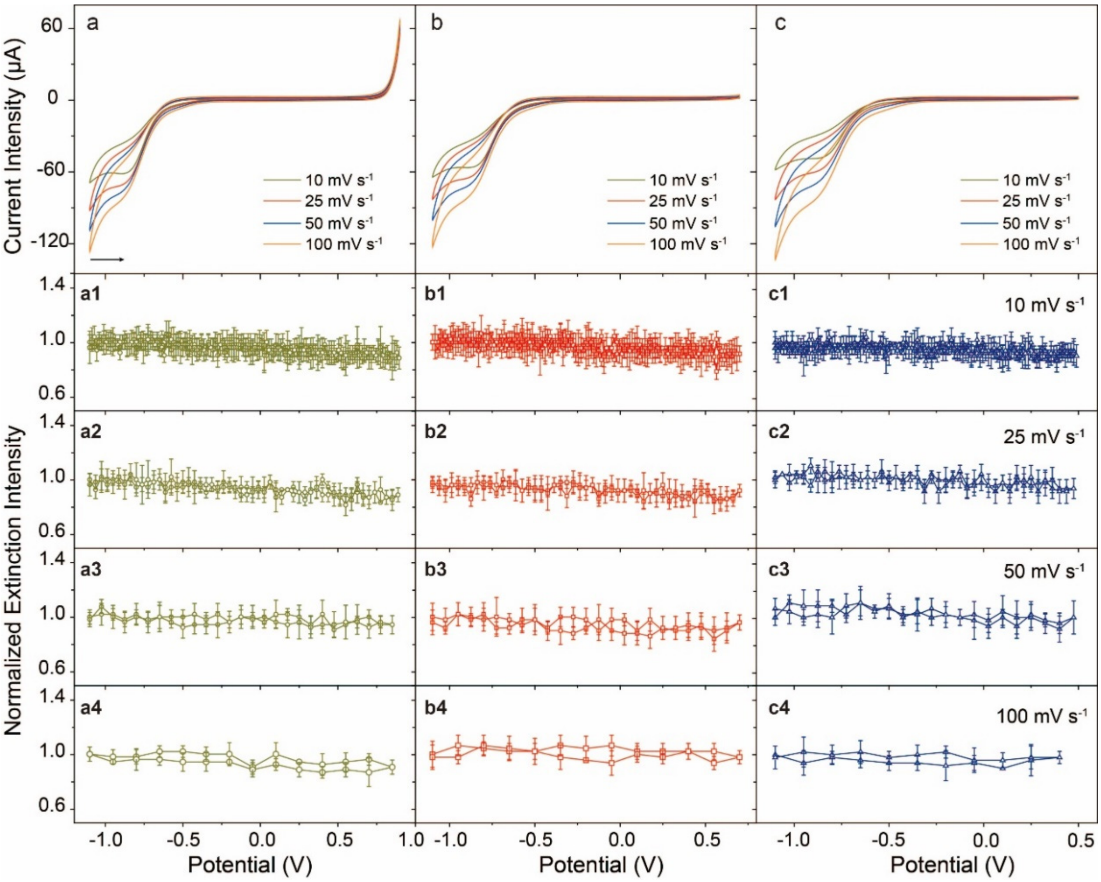

Supplementary Figure 22. CV curves for a bare ITO electrode in 0.10 KOH solution for oxygen evolution reaction in potential range from − 1.10 to 0.90 V *vs* Pt, at various scan rates 0.01 (a1), 0.025 (a2), 0.05 (a3) and 0.10 (a4) V s^{-1}; − 1.10 to 0.70 V *vs* Pt, at various scan rates 0.01 (b1), 0.025 (b2), 0.05 (b3) and 0.1 (b4) V s^{-1}; − 1.10 to 0.5 0V *vs* Pt, at various scan rates 0.01 (c1), 0.025 (c2), 0.05 (c3) and 0.10 (c4) V s^{-1}. The error bar represented the standard error during consecutive five potential scan. The corresponding CV curves at different potential scan range were shown in (a), (b) and (c), respectively.

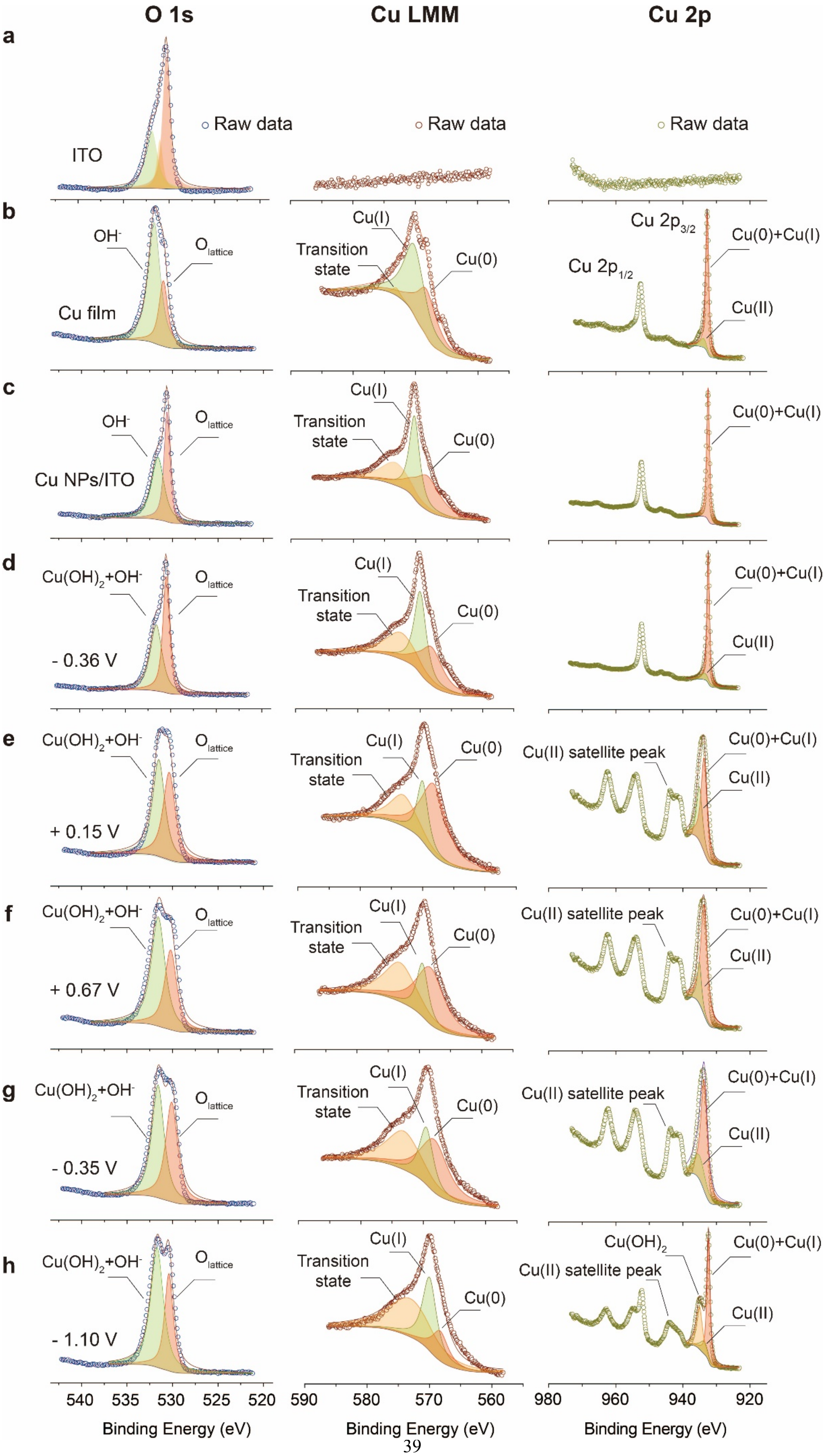

O 1s
Cu LMM
Cu 2p
a
b
c
d
e
f
g
h
Raw data
ITO
Cu film
Cu NPs/ITO
OH-
O lattice
Cu(OH)2+OH-
- 0.36 V
+ 0.15 V
+ 0.67 V
- 0.35 V
- 1.10 V
Cu(I)
Transition state
Cu(0)
Cu 2p3/2
Cu 2p1/2
Cu(0)+Cu(I)
Cu(II)
Cu(II) satellite peak
Cu(OH)2
540
535
530
525
520
590
580
570
560
980
960
940
920
Binding Energy (eV)

Supplementary Figure 23. Quasi in-situ XPS O 1s spectra, Cu Auger LMM and Cu 2p core level spectra obtained from a bare ITO electrode (a); a copper film (b); Cu NPs/ITO electrode (electrodeposition 150 cycles) (c); Cu NPs/ITO (150 cycles) after linear potential scan from – 1.10 V to – 0.36 V (d); Cu NPs/ITO (150 cycles) after linear potential scan from – 1.10 V to + 0.15 V (e); Cu NPs/ITO (150 cycles) after linear potential scan from – 1.10 V to + 0.90 V then to + 0.67 V (f); Cu NPs/ITO (150 cycles) after linear potential scan from – 1.10 V to + 0.90 V then to – 0.35 V (g); Cu NPs/ITO (150 cycles) after linear potential scan from – 1.10 V to + 0.90 V then to – 1.10 V (h), at a scan rate 0.01 V s^{-1}. Fitted peaks for individual species are shown with solid lines and annotations. O 1s includes the possible species: $Cu(OH)_2$,OH^-, $O_{lattice}$ (oxide lattice atoms). Cu Auger LMM includes the interfacial species: Cu(0) (568.2 eV); Cu(I) (570.2 eV); transition state (573.1 eV); Cu 2p includes the interfacial species: Cu(0) and Cu(I) (932.5 eV); Cu(II) (933.7 eV); $Cu(OH)_2$ (935.1 eV); Cu(II) satellite peaks (943.0 and 940.0 eV).

We further performed XPS to identify the interfacial species and to determine the chemical states of the Cu NPs/ITO under various polarization (Supplementary Figure 23). Supplementary Figure 23 displays XPS spectra of Cu NPs/ITO at various polarized potential and control samples (bare ITO and copper film). The XPS spectra reveals Cu 2p3/2 and Cu 2p1/2 peaks at 932.5 and 952.0 eV, respectively. In the high-resolution spectra, the Cu 2p3/2 at 933.7 eV, 935.1 eV and a series of satellites 943.0 and 940.0 eV are assigned to Cu(II); and the peak at 932.0 eV is correlated to Cu(0) and Cu(I).[31-32]

Despite some overlapping, Cu(0) and Cu(I) can still be distinguished from the Cu Auger LMM spectra. As revealed in Supplementary Figure 23, Cu Auger LMM of Cu film and fresh electrodeposited Cu NPs both exhibited the presence of Cu(I) component located at 570.2 eV, which might be attributed to the fast oxidation of metallic Cu during transferring to the XPS chamber in air.[32-33]

The surface species changes during dynamic electrochemical scan was shown in Supplementary Figure 23 (e-h). After linear potential scan from – 1.10 V to – 0.36 V in 0.1 M KOH, the amounts of Cu(I) species increased *ca.* 20 %. With further enhancement of anodic polarization at + 0.15 V, the emergence of Cu(II) (933.7 eV) and Cu(II) satellite peaks indicated the presence of CuO and $Cu(OH)_2$ in the passive region (from – 0.10 V to + 0.20 V). Interestingly, comparing the interfacial composition at + 0.15 V (passive region) and + 0.67 V (peak b), negligible change was observed, thus excluding the possible fluctuation of extinction intensity in peak b induced by interfacial phase transitions. Therefore, the corresponding extinction intensity dynamics in peak b was attributed to the oxygen multi-diffusion behavior.

In oxygen accelerated region (– 1.10 V), the reduction of oxygen resulted in remarkable adsorption of hydroxyl groups on the surface, that confirmed by the obvious increase of the intensity of $Cu(OH)_2$ and OH^- in O 1s peak (531.9 eV). In summary, the above quasi in-situ XPS analysis reveals detailed phase transitions under various polarization and provides direct chemical information to corroborate our electrochemical experiments.

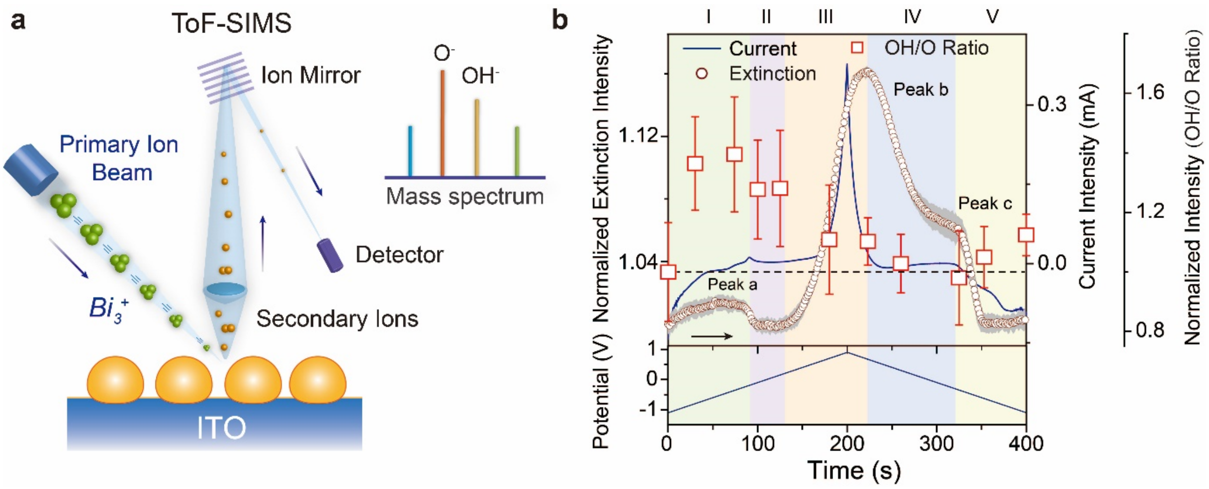

Supplementary Figure 24. (a) Schematic of quasi in-situ ToF-SIMS monitoring the interfacial OH/O ratio at the Cu NPs/ITO electrode surface. The high-energy primary ion beam Bi_3^+ bombards the electrode surface causes emission of secondary ions, which can be accelerated and captured by a specific polarized electric field and then transferred to a time-of-flight mass analyzer. It can provide specific compound identification of elements and molecules on a surface. (b) The representative dynamic extinction trajectory (○), current curve (–) and normalized OH/O ratio (□) for OER on a plasmonic Cu NPs electrode scanned from – 1.10 to + 0.90 V at a scan rate 0.01 V s^{-1}. The dash line is used to guide the eye for comparison the OH/O ratio. The detailed negative mass spectra of Cu NPs/ITO electrode at various potential are shown in Supplementary Figure 25.

We further performed Time-of-Flight Secondary Ion Mass Spectrometry (ToF-SIMS) to obtain precise interfacial elements or molecules information. ToF-SIMS is a sensitive surface technique that enables to distinguish interfacial species with the ability to differentiate a proton between two similar species.[34-35]ToF-SIMS results indicated a distinct fluctuation of interfacial OH/O ratio under various polarization (Supplementary Figure 24 and 25):

(1) In peak a, a gradual OH/O ratio increase companied by slight decrease was observed during potential range – 1.10 V to – 0.10 V, which was of the same evolution trend as predict by electrochemical measurements. The dynamic of extinction intensity in peak a could be attributed to interfacial Cu oxides transitions.

(2) In passive region (potential from – 0.10 V to + 0.20 V), the OH/O ratio maintains a steady state, which provides another direct molecular evidence to confirm the interfacial stability.

(3) In peak b (potential from + 0.20 to + 0.90 V then to + 0.67 V), no obvious fluctuation of OH/O ratio was observed. This helps to exclude the possibility that the interfacial oxide transitions lead to remarkable extinction intensity changes. Therefore, the dramatic fluctuation of extinction intensity in peak b can only be correlated with oxygen evolution

reaction.

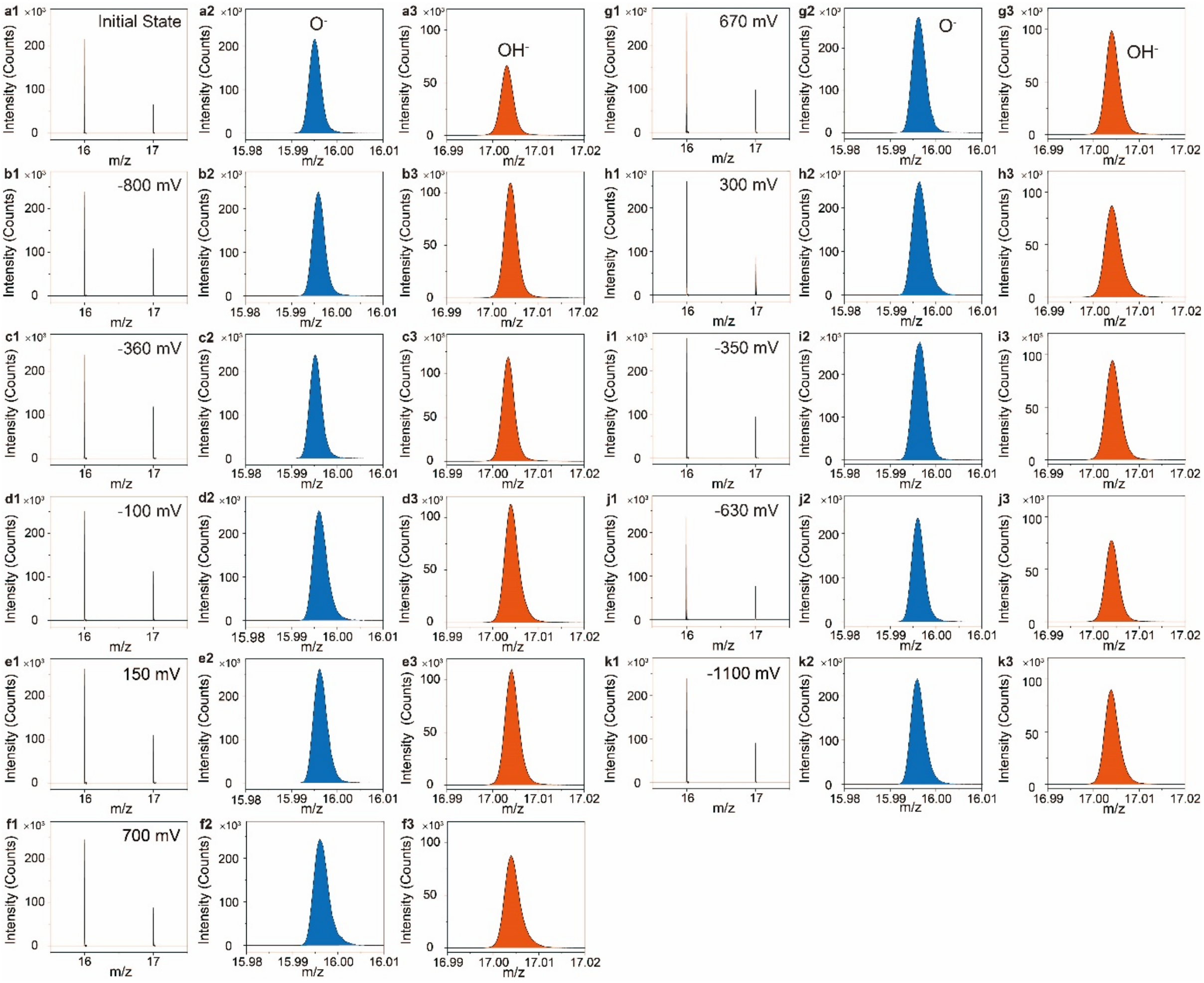

Supplementary Figure 25. Negative ion mass spectra of Cu NPs/ITO electrode at various polarized potential: (a1) the initial state of Cu NPs/ITO electrode; (b1) after linear potential scan from – 1.10 V to – 0.80 V; (c1) after linear potential scan from – 1.10 V to – 0.36 V; (d1) after linear potential scan from – 1.10 V to – 0.10 V; (e1) after linear potential scan from – 1.10 V to + 0.15 V; (f1) after linear potential scan from – 1.10 V to + 0.70 V; (g1) after linear potential scan from – 1.10 V to + 0.90 V then to + 0.67 V; (h1) after linear potential scan from – 1.10 V to + 0.90 V then to + 0.30 V; (i1) after linear potential scan from – 1.10 V to + 0.90 V then to – 0.35 V; (j1) after linear potential scan from – 1.10 V to + 0.90 V then to – 0.63 V; (k1) after linear potential scan from – 1.10 V to + 0.90 V then to – 1.10 V. (a2-k2) and (a3-k3) are the enlarged view of O^- and OH^- corresponding to various potential (a1-k1), respectively. The OH/O ratio was calculated by comparison the integral area between OH^- and O^-.

Supplementary Note 7: Concentration Overpotential for Oxygen Evolution Reaction

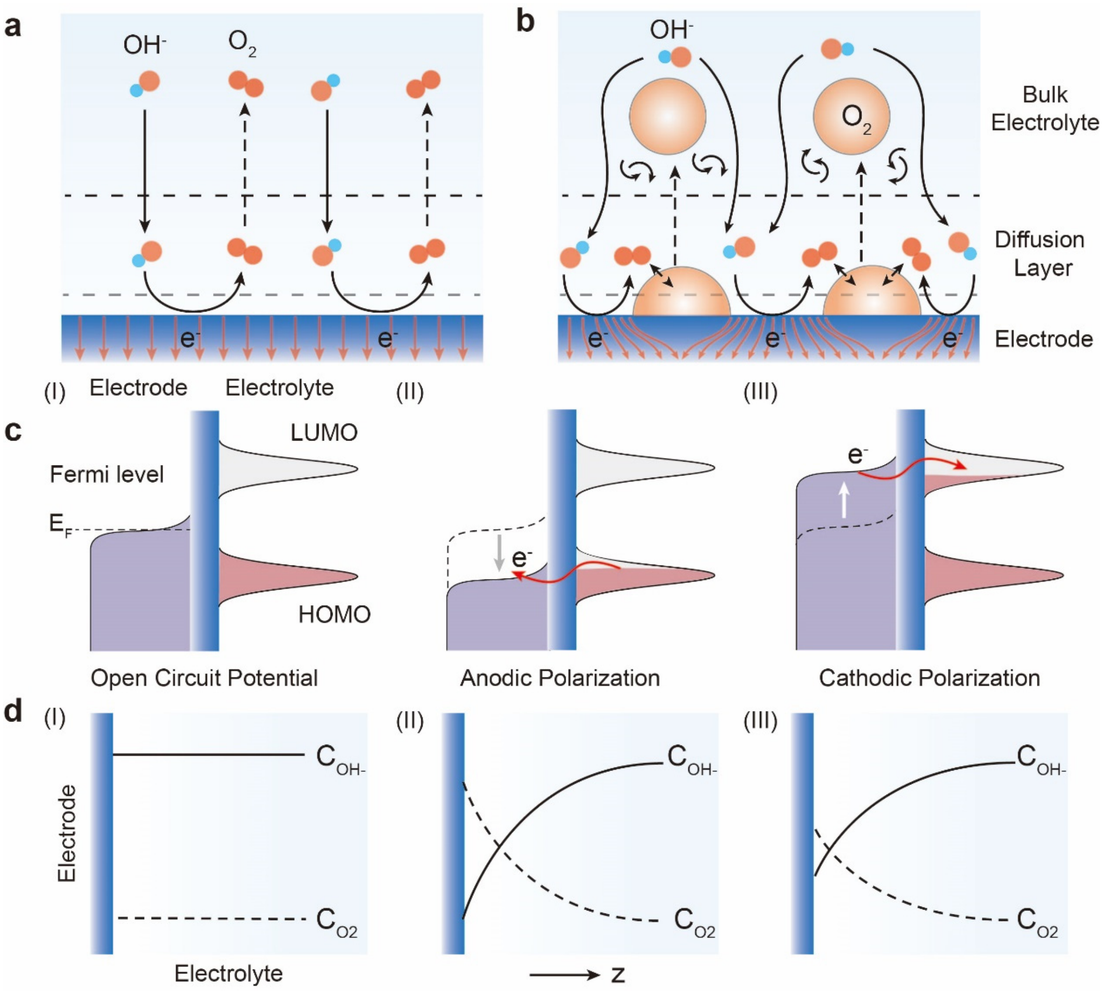

Supplementary Figure 26. Schematic for the diffusion of reactant and product and electrochemical processes during oxygen evolution reaction at the electrode-electrolyte interface in absence (a) and presence (b) surface gas bubble at the electrode surface. (c) The corresponding energy level for the oxygen evolution electrode at open circuit potential (I), anodic polarization (II) and cathodic polarization (III). (d) The concentration distribution of OH^- and O_2 in the diffusion layer at open circuit potential (I), anodic polarization (II) and cathodic polarization (III).

The difference in the concentrations of electrochemical reactants and products at the electrode-electrolyte interface compared those in the bulk electrolyte contributed to the generation of the concentration overpotential loss (η_c) (Supplementary Figure 26). In order to quantify this loss during oxygen evolution reaction, a one-dimension diffusion model was developed. The deviation of the concentration potential (η_c) as a function of current density (i) for the oxygen evolution at the electrode-electrolyte interface followed the similar derivation across SiO_x nanomembrane overlayer.[36-37] The current derivation for the oxygen evolution reaction (OER), $4OH^- - 4e^- \rightarrow O_2 + 2H_2O$, offered a general expression η_c for reaction O + ne^-

→ R.

$$\eta_c = E(z=0) - E(z=d) \tag{7-1}$$

where E was the potential at distance z from the electrode-electrolyte interface (z was vertical coordinate), d was the height of the oxygen diffusion boundary from the electrode surface. According to the Nernst Equation[38], the potential E could be expressed as:

$$\mathrm{E} = E^{\circ\prime}_{OH^-/O_2} + \frac{RT}{nF} ln \frac{C^4_{OH^-}}{C_{O_2}} \tag{7-2}$$

where T is absolute temperature, R is the gas constant, F is the Faraday's constant and n was the electron transfer number (n = 4). Moreover, the formal potential $E_{OH^-/O2}{}^{\circ\prime} = E_{OH^-/O2}{}^{\circ} + \beta$, where $E_{OH^-/O2}{}^{\circ}$ was the standard reduction potential and β was the contribution from the activity coefficients for both OH^- and O_2. Based on the diffusion equations, the diffusive fluxes for OH^- and O_2 were described by Fick's Law, assuming without obvious gas bubble evolution.[39] The concentration gradient closed to the electrode-electrolyte interface prompted these species diffusion across the diffusion layer. Hence, the diffusive fluxes of OH^- and O_2 were expressed as:

$$J_{OH^-} = -D_{OH^-} \frac{dC_{OH^-}}{dz} \tag{7-3}$$

$$J_{O_2} = -D_{O_2} \frac{dC_{O_2}}{dz} \tag{7-4}$$

In order to describe the solubility of OH^- and the oversaturation of O_2 in the diffusion layer, the partition coefficient Φ was defined as the ratio between the solubility of OH^- and the oversaturation of O_2 in the diffusion layer and their concentration in bulk electrolyte ($C_{OH-,\,b}$ and $C_{O2,\,b}$):

$$\Phi(OH^-) = \frac{C_{OH^-,d}}{C_{OH^-,b}} \tag{7-5}$$

$$\Phi(O_2) = \frac{C_{O_2,d}}{C_{O_2,b}} \tag{7-6}$$

Based on the Faraday's law and equations 7-5 and 7-6, the current density for OH^- and O_2 was estimated, respectively:

$$i_{OH^-} = -2FD_{OH^-} \frac{\Phi(OH^-)C_{OH^-,b} - C_{OH^-,s}}{d} \tag{7-7}$$

$$i_{O_2} = -4FD_{O_2} \frac{\Phi(O_2)C_{O_2,b} - C_{O_2,s}}{d} \tag{7-8}$$

where $C_{OH-, s}$ and $C_{O2, s}$ was denoted as the concentration of OH^- and O_2 at the electrode-electrolyte interface, respectively, d was the thickness of a Nernst diffusion layer (a boundary layer). Under the mass transfer limited conditions, the limiting current for OH^- and O_2 was expressed as:

$$i_{l,OH^-} = -2FD_{OH^-}\frac{\Phi(OH^-)C_{OH^-,b}}{d} \tag{7-9}$$

$$i_{l,O_2} = -4FD_{O_2}\frac{\Phi(O_2)C_{O_2,b}}{d} \tag{7-10}$$

According to the Bulter-Volmer equation, cathodic and anodic branches under mass transport limitation was written as:

$$i_{l,OH^-} = i_0\left[\Phi(OH^-)exp\left(\frac{(1-\alpha)ne\eta}{kT}\right)\right] \tag{7-11}$$

$$i_{l,O_2} = i_0\left[\Phi(O_2)exp\left(\frac{-\alpha ne\eta}{kT}\right)\right] \tag{7-12}$$

where n was the number of electrons transferred during evolution reaction which was 4 in our case, i_0 here denoted the exchange current density, α was the charge transfer coefficient, η was the overpotential corresponding to the thermodynamic equilibrium, both $C_{OH-, s}$ and $C_{O2, s}$ were zero at the electrode surface and the $i_{l, OH-}$ and $i_{l, O2}$ were the limiting current density of OH^- oxidation (anodic polarization) and O_2 reduction (cathodic polarization), respectively. During the oxygen evolution reaction under anodic polarization, $i_{l, OH-}$ was the main contributor for the current density in which $i_{l, O2}$ was neglected. Hence, we obtained the equations to estimate $C_{OH-, s}$ and $C_{O2, s}$, respectively.

$$C_{OH^-,s} = \Phi(OH^-)C_{OH^-,b}\left(1 - \frac{i}{i_{l,OH^-}}\right) \tag{7-13}$$

$$C_{O_2,s} = \Phi(O_2)C_{O_2,b}\left(1 - \frac{i}{i_{l,O_2}}\right) \tag{7-14}$$

Combining equations 7-11, 7-12 and 7-2, the potential at the electrode interface, $E(z = 0)$ and the potential in diffusion layer, $E(z = d)$ were obtained:

$$E(z = 0) = E^{\circ\prime}_{OH^-/O_2} + \frac{RT}{nF}ln\frac{\left(\Phi(OH^-)C_{OH^-,b}\left(1-\frac{i}{i_{l,OH^-}}\right)\right)^4}{\Phi(O_2)C_{O_2,b}\left(1-\frac{i}{i_{l,O_2}}\right)} \tag{7-15}$$

$$E(z = d) = E^{\circ\prime}_{OH^-/O_2} + \frac{RT}{nF}ln\frac{C^4_{OH^-,b}}{C_{O_2,b}} \tag{7-16}$$

When we substituted the equations 7-15 and 7-16 into equation 7-1, the concentration overpotential, η_c induced by the oversaturation of oxygen was expressed as:

$$\eta_c = \frac{RT}{nF} ln \left(\Phi(OH^-) \left(1 - \frac{i}{i_{l,OH^-}} \right) \right)^4 \left(\Phi(O_2) \left(1 - \frac{i}{i_{l,O_2}} \right) \right)^{-1} \quad (7\text{-}17)$$

In order to understand the influence from saturation of oxygen during anodic polarization on the gas evolution reaction, the linear scan voltammetry (LSV) was precisely modeled and the bulk mass transport losses (η_m), kinetic losses (η_k), electrolyte ohmic losses (η_o) and concentration overpotential (η_c) were considered.

$$\eta_t = \eta_m + \eta_k + \eta_o + \eta_c \quad (7\text{-}18)$$

Here, the ohmic *iR* losses (η_o) had been corrected in LSV curves based on electrochemical impedance spectroscopy. On the basis of the same electrode geometry and active catalytic sites, both η_k and η_m were the same for the electrochemical measurements and electrochemical extinction measurements. Hence, the different overpotential for electrochemical measurements and electrochemical extinction measurements were mainly contributed from the concentration overpotential. The LSV curves in both electrochemical and in-situ extinction measurements were fitted by a nonlinear fitting (Supplementary Figure 27). Interestingly, when the increased current density approached to the limiting current density under enhanced anodic polarization, the limitation of the model could be evident. It offered evidence for the presence diffusion limitation under anodic polarization. According to the fitting parameter, the difference concentration overpotential (η_c) *ca.* 0.20 V *vs.* Pt between in-situ extinction measurement and electrochemical measurements was estimated.

Under practical electrochemical measurements, the total current density was composed by the sum of convention, migration, diffusion and electric double layer charging. In general, it was expressed by the sum of the Faradaic and non-Faradic portion for the total current density:

$$i_t = i_c + i_m + i_d + i_{ec} \quad (7\text{-}19)$$

$$i_t = i_F + i_{nF} \quad (7\text{-}20)$$

During the electrochemical measurements, the record current included both the Faradaic and non-Faradic current ($i_F + i_{nF}$). However, during in-situ extinction measurements, the fluctuation of extinction intensity directly represented the oxygen concentration fluctuation at the electrode-electrolyte interface. Therefore, the correlation between the extinction intensity and the Faradaic current intensity could be established, so we could use the extinction intensity to denote the Faradaic current intensity (i_F). In an anodic polarization, oxygen evolution at the

electrode surface was proceeding during persistent polarization, the relative measured current density was given by:

$$i_e = \mathrm{nF}\frac{D_{O_2}}{d}(C^{e}_{O2,s} - C_{O2,b}) = i_F + i_{nF} \tag{7-21}$$

$$i_{ext} = \mathrm{nF}\frac{D_{O_2}}{d}(C^{ext}_{O2,s} - C_{O2,b}) = i_F \tag{7-22}$$

where i_e and i_{ext} were the measured current during electrochemical and in-situ electrochemical extinction measurements, respectively, d was the thickness of the diffusion layer, $C_{O2,\,b}$ was the bulk concentration, D_{O2} was the diffusion coefficient of the dissolved oxygen in the electrolyte, F is Faraday's constant, n was the electron number involved in the reaction, $C_{O2,\,s}{}^{e}$ and $C_{O2,\,s}{}^{ext}$ were the apparent oxygen concentration at the electrode surface measured by electrochemical and in-situ extinction measurements, respectively. Due to the influence from the non-Faradaic current on the measured oxygen concentration, it could be found that $C_{O2,\,s}{}^{e} > C_{O2,\,s}{}^{ext}$. The accumulation of oxygen in the diffusion layer contributed to the shift of electrode potential (concentration polarization), which was simplified expressed by a Nernstian equation:[40]

$$\eta_c = 2.3\frac{RT}{nF}log\frac{C_{O2,s}}{C_{O2,b}} \tag{7-23}$$

Considering $C_{O2,\,s}{}^{e} > C_{O2,\,s}{}^{ext}$, hence, the η_c obtained in electrochemical procedures was larger than that measured during in-situ extinction measurement, which gave the distinct evidence for the difference in the measured concentration polarization potential and relative onset potential. Most importantly, the above analysis demonstrated that in-situ extinction measurement provided direct information related to Faradaic process without the interference from the non-Faradaic components, which possessed the capacity to precisely determine the relevant parameters in gas evolution reactions such as concentration overpotential and onset potential etc.

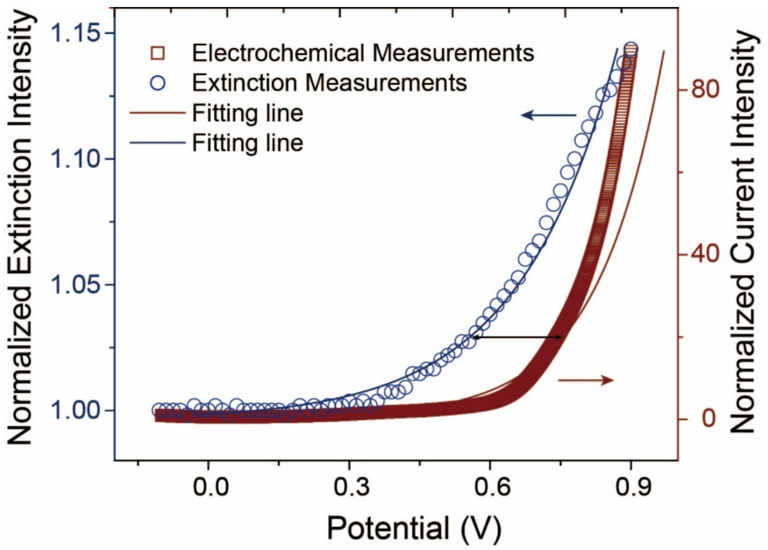

Supplementary Figure 27. Comparison the difference of concentration overpotential between electrochemical measurement and in-situ extinction spectroelectrochemistry. The fitting line were based on the proposed model and it was suitable for fitting the data at low and intermediate current density.

Supplementary Note 8: FDTD Simulation for Oxygen Evolution at the Electrode-Electrolyte Interface

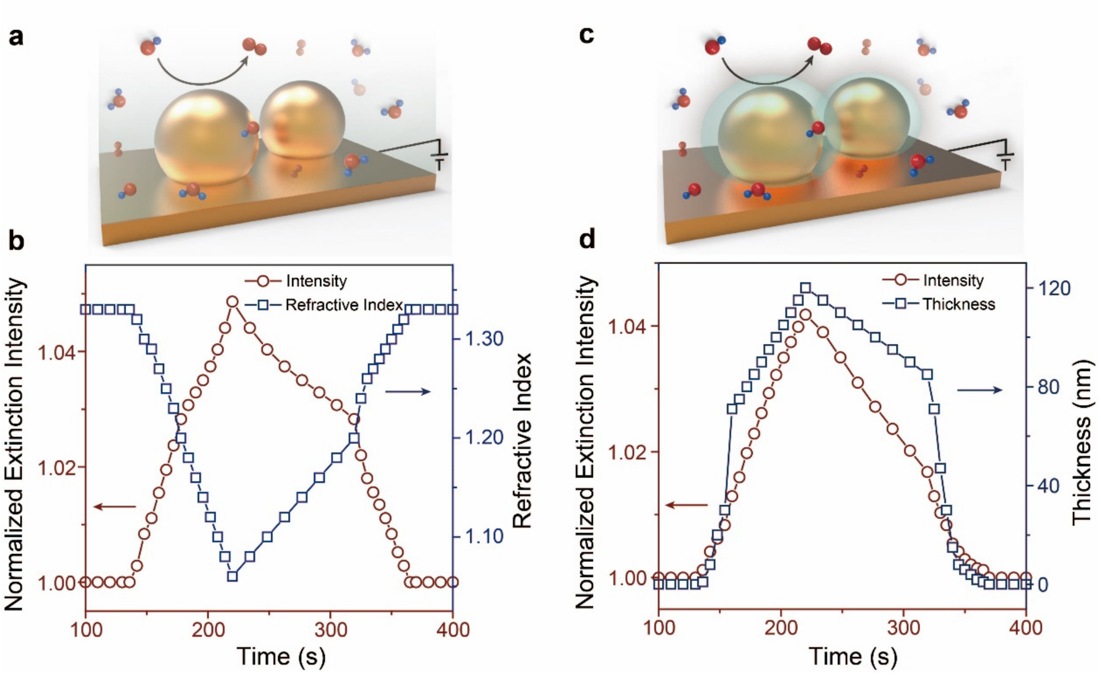

Supplementary Figure 28. (a) Schematic illustration of the electrogenerated oxygen induced dielectric environment change around the plasmonic nanoparticles. (b) Simulated normalized extinction intensity as a function of environment refractive index from 1.33 to 1.06. (c) Schematic illustration of the electrogenerated oxygen shell around the plasmonic nanoparticles. (d) Simulated normalized extinction intensity as a function of the thickness of oxygen shell from 0 to 120 nm. In order to simulated the influence of oxygen evolution on the extinction intensity, we established two models to simulate the different thickness of oxygen shell and various environment refractive index around plasmonic nanoparticles, respectively. In all cases, the extinction intensity enhanced with increasing the thickness of oxygen shell and decreasing of refractive index of the surrounding dielectric medium, respectively. Both simulated extinction intensity evolutions were agreed well with in-situ extinction measurements (Figure 2e), further indicated the capability of plasmonic single wavelength strategy in revealing details on the interfacial dynamics during oxygen evolution reaction.

Supplementary Note 9: Numerical Simulation for Oxygen Diffusion at the Electrode-Electrolyte Interface

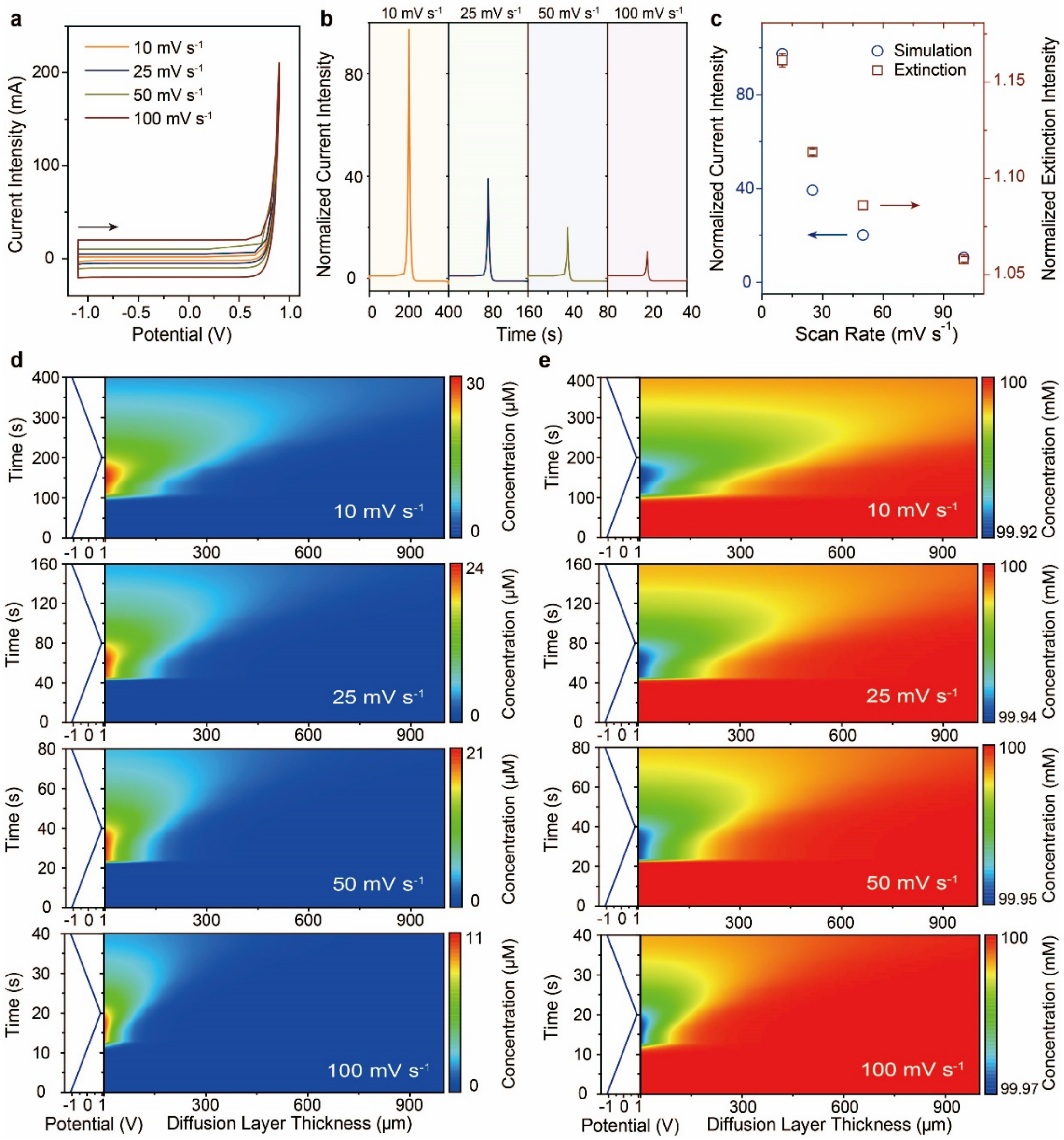

Supplementary Figure 29. (a) Numerical simulation CV curves for oxygen evolution reaction in potential range from − 1.10 to 0.90 V, at various scan rates 0.01, 0.025, 0.05 and 0.10 V s^{-1}. (b) The normalized temporal current intensity for oxygen evolution reaction at various scan rates 0.01, 0.025, 0.05 and 0.10 V s^{-1}. In order to eliminate the charging effect on the evaluation of oxygen evolution reaction, the charging current at each scan rate serves as the reference for normalization. (c) The comparison between experimental extinction intensity and numerical simulation current intensity dependent on the square root of the scan rate. Numerical simulation of potential dependent evolution of oxygen diffusion layer (contour plots) (d) and hydroxyl (e) along the axial direction of the EEIs at various scan rates 0.01, 0.025, 0.05 and 0.10 V s^{-1}.

In order to reveal the oxygen and hydroxide distribution at the electrode-electrolyte interface, one-dimension finite element modelling was established. Assuming the presence of a large

quantity supporting electrolyte to increase the conductivity of the electrolyte without any perturbance on the interfacial electrochemical reaction. Hence, the resistance of the solution was relatively low and the induced electric field was negligible. To describe the interfacial mass transfer of the reactant and product species involved in the electrochemical reaction, we adopted Fick's 2nd law to illustrate the chemical mass transport of the electroactive species OH^- and O_2:[41]

$$\frac{\partial c_i}{\partial t} = \nabla \cdot (D_i \nabla c_i) \tag{8-1}$$

At the initial stage, a uniform concentration distribution at the bulk electrolyte (x = *L*) and no product was present in bulk. At the electrode surface (x = 0), the reactant OH^- lost on electron to form 0.25 equivalent oxygen. The electrochemical interfacial reaction was expressed as the reductive form:

$$1/4\ O_2 + 1/2\ H_2O + e^- \rightarrow OH^- \tag{8-2}$$

The current density of this reaction was calculated by the Bulter-Volmer equation for an oxidation:[11]

$$i_a = nFk_0\left(c_{OH^-} exp\left(\frac{(n-\alpha)F\eta}{RT}\right) - c_{O_2} exp\left(\frac{-\alpha F\eta}{RT}\right)\right) \tag{8-3}$$

where F is the Faraday's constant (96485 C mol^{-1}), k_0 was the heterogeneous rate constant of the reaction (5e-16 m s^{-1}, which was taken from the rate determining step for formation of the peroxide intermediate HOO during oxygen evolution reaction)[42], α was the cathodic transfer coefficient (0.5) and η was the overpotential at the working electrode relative to the equilibrium potential of the redox couple of OH^- and O_2. Furthermore, based on Faraday's law, the electrode surface boundary condition was drawn in which the flux of the reactant and product species ($\boldsymbol{N}_i$) were proportional to their current density (i_a):

$$-\mathbf{n} \cdot \boldsymbol{N}_i = \frac{\nu_i i_a}{nF} \tag{8-4}$$

Considering the oxygen evolution reaction at the Cu NPs/ITO electrode was performed at relatively large overpotential under kinetic control, the polarized current was assumed to be independent of the thermodynamic performance of electrocatalysts. Hence, with enhancement of the anodic polarization, a diffusional concentration profile was developed which showed dynamic behavior as a function of distance from the electrode surface. Based on the numerical simulation, the dynamic oxygen and hydroxide concentration profile during dynamic potential

and the corresponding cyclic voltammetry curves scan was identified shown in Supplementary Figure 29. With the elimination of the charging current through the normalization the total current intensity respecting to the capacitive charging current, the temporal current intensity indicated a similar trend with the in-situ extinction voltammetry measurements as a function of potential scan rate. It demonstrated that the extinction voltammetry could directly reflect the dynamic oxygen evolution at the electrode-electrolyte interface without interference from the non-Faradaic processes (Supplementary Figure 29, a-c). Moreover, the numerical simulation of the potential-dependent concentration profiles of both reactant (hydroxide) and products (oxygen) revealed that the conspicuous confinement for oxygen in diffusion layer at high potential scan rate (Supplementary Figure 29, d-e) and in line with the in-situ extinction measurements (Figure 4, in main text). Both these results further confirmed that the in-situ electrochemical extinction measurements enabled the real time monitoring the dynamic diffusion profiles of oxygen during dynamic potential scan without complex optical marker and corroborated the oxygen diffusion limiting effect on oxygen evolution reaction at the electrode-electrolyte interface, which was hampered in conventional electrochemical measurements.

Supplementary Note 10: Elimination of Interfacial Dissolved Oxygen at the Electrode-Electrolyte Interfaces

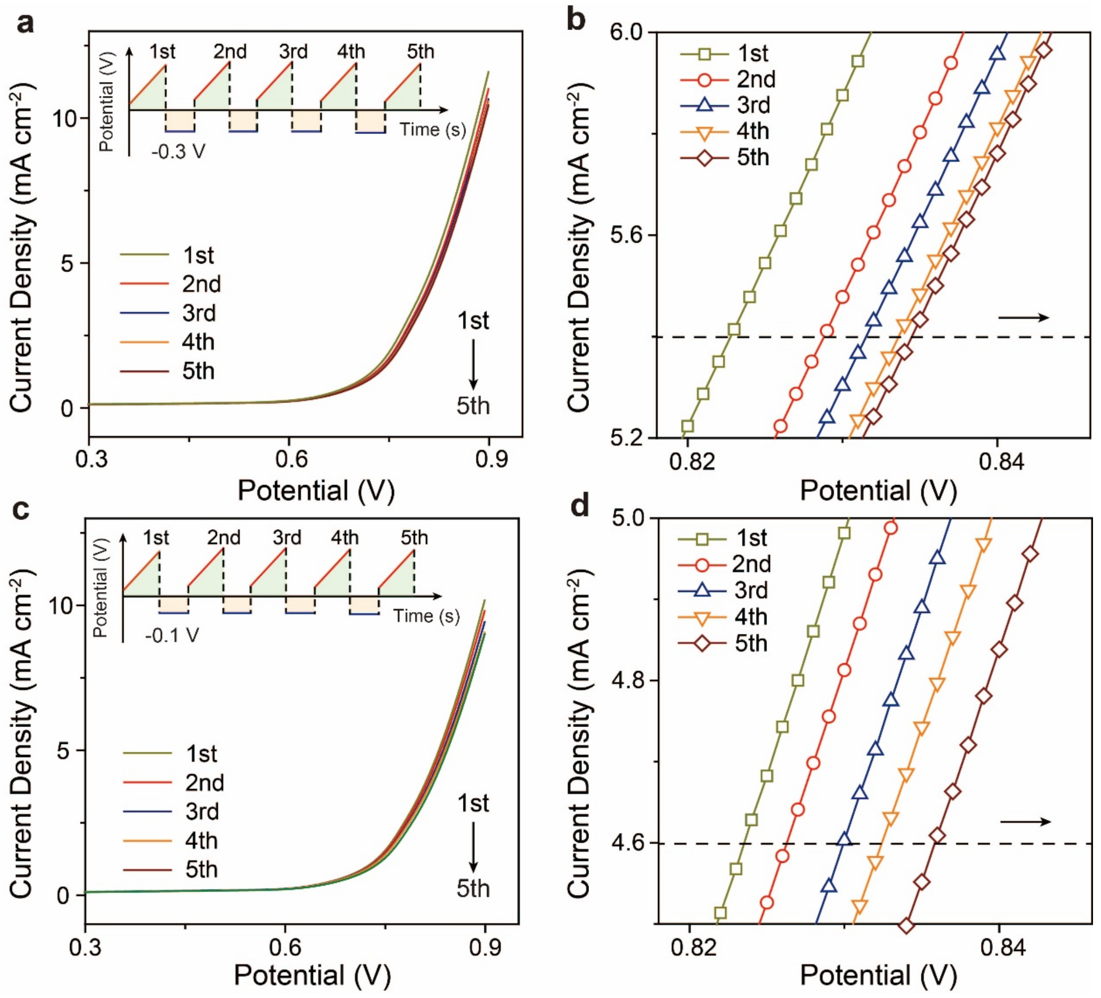

Supplementary Figure 30. Linear sweep voltammetry curves of Cu electrode followed reduction potential multi-pulse (a − 0.30 V and c − 0.10 V) in 0.10 M KOH solution at a scan rate of 0.01 V s^{-1}. The enlarged cathodic shift of the oxygen evolution potential as consecutive reduction potential pulses (b − 0.30 V and d − 0.10 V).

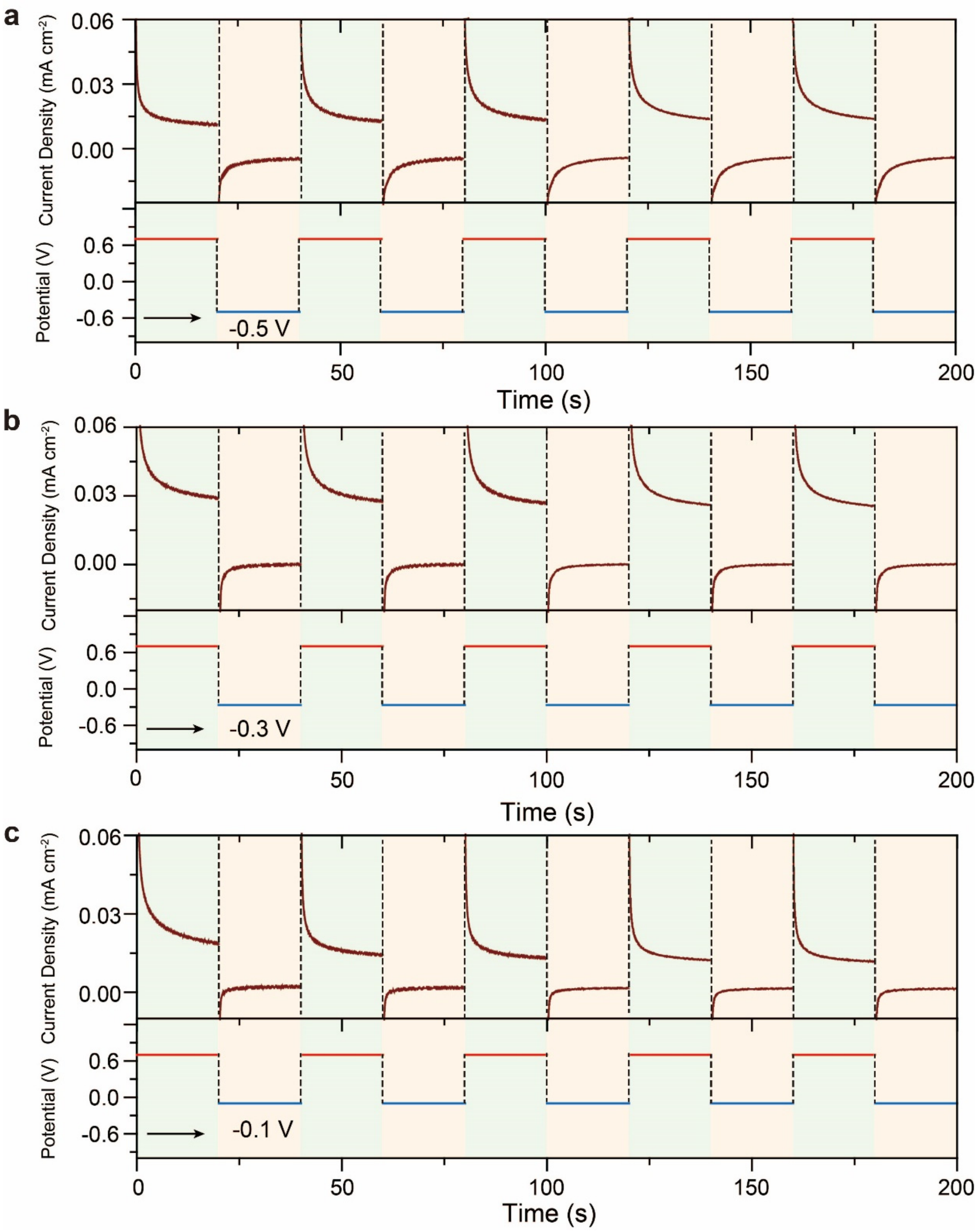

Supplementary Figure 31. Chronoamperometry curves on Cu electrode obtained from the programmed potential scan: (a) anodic potential 0.70 V, step time 20 s; cathodic potential − 0.50 V, step time 20 s; (a) anodic potential 0.70 V, step time 20 s; cathodic potential − 0.30 V, step time 20 s; (a) anodic potential 0.70 V, step time 20 s; cathodic potential − 0.10 V, step time 20 s.

Supplementary References

(1) Steinhauer, C.; Jungmann, R.; Sobey, T. L.; Simmel, F. C.; Tinnefeld, P., DNA origami as a nanoscopic ruler for super-resolution microscopy. *Angew. Chem., Int. Ed.* **2009,** *48*, 8870-8873.

(2) Wang, J.-G.; Hua, X.; Li, M.; Long, Y.-T., Mussel-inspired polydopamine functionalized plasmonic nanocomposites for single-particle catalysis. *ACS Appl. Mater. Interfaces* **2017,** *9*, 3016-3023.

(3) Johnson, P. B.; Christy, R. W., Optical constants of the noble metals. *Phys. Rev. B* **1972,** *6*, 4370-4379.

(4) Ribbing, C. G.; Roos, A., Copper Oxides (Cu_2O, CuO). *Handbook of Optical Constants of Solids*, Palik, E. D., Ed. Academic Press: Boston, 1998; 875-882.

(5) Zhang, J.; Lu, Z. H.; Wang, L. J., Precision refractive index measurements of air, N_2, O_2, Ar, and CO_2 with a frequency comb. *Appl. Opt.* **2008,** *47*, 3143-3151.

(6) Plieth, W., *Electrochemistry for materials science*. Elsevier: 2008, 195-229.

(7) Pei, A.; Zheng, G.; Shi, F.; Li, Y.; Cui, Y., Nanoscale nucleation and growth of electrodeposited lithium metal. *Nano Lett.* **2017,** *17*, 1132-1139.

(8) Winand, R., Electrocrystallization: Fundamental considerations and application to high current density continuous steel sheet plating. *J. Appl. Electrochem.* **1991,** *21*, 377-385.

(9) El-Deab, M. S., On the preferential crystallographic orientation of Au nanoparticles: Effect of electrodeposition time. *Electrochim. Acta* **2009,** *54*, 3720-3725.

(10) Wang, J.; Cao, X.; Li, L.; Li, T.; Wang, R., Electrochemical seed-mediated growth of surface-enhanced Raman scattering active Au(111)-like nanoparticles on indium tin oxide electrodes. *J. Phys. Chem. C* **2013,** *117*, 15817-15828.

(11) Bard, A. J., Faulkner, L. R., *Electrochemical Methods, Foundamentals and Applications*. *John wiley and Sons Inc*: *New York*, 2001; Vol. 482.

(12) Emanuel, A.; Olander, D. R., Diffusion coefficients of copper sulfate in water and water in n-butyl alcohol. *J. Chem. Eng. Data* **1963,** *8*, 31-32.

(13) Hillson, P. J., The mechanism of the reaction at a Cu/Cu^{2+} electrode. *Trans. Faraday Soc.* **1954,** *50*, 385-393.

(14) Finot, M. O.; Braybrook, G. D.; McDermott, M. T., Characterization of electrochemically deposited gold nanocrystals on glassy carbon electrodes. *J. Electroanal. Chem.* **1999,** *466*, 234-241.

(15) Praig, V. G.; Piret, G.; Manesse, M.; Castel, X.; Boukherroub, R.; Szunerits, S., Seed-mediated electrochemical growth of gold nanostructures on indium tin oxide thin films. *Electrochim. Acta* **2008,** *53*, 7838-7844.

(16) Vitos, L.; Ruban, A. V.; Skriver, H. L.; Kollár, J., The surface energy of metals. *Surf. Sci.* **1998,** *411*, 186-202.

(17) Pattadar, D. K.; Zamborini, F. P., Effect of size, coverage, and dispersity on the potential-controlled Ostwald ripening of metal nanoparticles. *Langmuir* **2019,** *35*, 16416-16426.

(18) Bogdanowicz, R.; Ryl, J.; Darowicki, K.; Kosmowski, B., Ellipsometric study of oxide formation on Cu electrode in 0.1 M NaOH. *J. Solid State Electrochem.* **2009,** *13*, 1639-1644.

(19) Abd el Haleem, S. M.; Ateya, B. G., Cyclic voltammetry of copper in sodium hydroxide solutions. *J. Electroanal. Chem. Interfacial Electrochem.* **1981,** *117*, 309-319.

(20) He, M.; Li, C.; Zhang, H.; Chang, X.; Chen, J. G.; Goddard, W. A.; Cheng, M.-j.; Xu, B.; Lu, Q., Oxygen induced promotion of electrochemical reduction of CO_2 via co-electrolysis. *Nat. Commun.* **2020,** *11*, 3844.

(21) Sheng, W.; Myint, M.; Chen, J. G.; Yan, Y., Correlating the hydrogen evolution reaction activity in alkaline electrolytes with the hydrogen binding energy on monometallic surfaces. *Energy Environ. Sci.* **2013,** *6*, 1509-1512.

(22) Zheng, J.; Sheng, W.; Zhuang, Z.; Xu, B.; Yan, Y., Universal dependence of hydrogen oxidation and evolution reaction activity of platinum-group metals on pH and hydrogen binding energy. *Sci. Adv.* **2016,** *2*, e1501602.

(23) Gao, R.; Edwards, M. A.; Qiu, Y.; Barman, K.; White, H. S., Visualization of hydrogen evolution at individual platinum nanoparticles at a buried interface. *J. Am. Chem. Soc.* **2020,** *142*, 8890-8896.

(24) Tao, H. B.; Xu, Y.; Huang, X.; Chen, J.; Pei, L.; Zhang, J.; Chen, J. G.; Liu, B., A general method to probe oxygen evolution intermediates at operating conditions. *Joule* **2019,** *3*, 1498-1509.

(25) Link, S.; El-Sayed, M. A., Size and temperature dependence of the plasmon absorption of colloidal gold nanoparticles. *J. Phys. Chem. B* **1999,** *103*, 4212-4217.

(26) Bohren, C. F., Huffman, D. R., Absorption and scattering of light by small particles. John Wiley & Sons, 2008; 82-129.

(27) Kumar, A.; Kim, S.; Nam, J.-M., Plasmonically engineered nanoprobes for biomedical applications. *J. Am. Chem. Soc.* **2016,** *138*, 14509-14525.

(28) Mayer, K. M.; Hafner, J. H., Localized surface plasmon resonance sensors. *Chem. Rev.* **2011,** *111*, 3828-3857.

(29) Anderson, L. J. E.; Mayer, K. M.; Fraleigh, R. D.; Yang, Y.; Lee, S.; Hafner, J. H., Quantitative measurements of individual gold nanoparticle scattering cross sections. *J. Phys. Chem. C* **2010,** *114*, 11127-11132.

(30) Gerislioglu, B.; Dong, L.; Ahmadivand, A.; Hu, H.; Nordlander, P.; Halas, N. J., Monolithic metal dimer-on-film structure: new plasmonic properties introduced by the underlying metal. *Nano Lett.* **2020,** *20*, 2087-2093.

(31) Yang, C.-J.; Lu, F.-H., Shape and size control of Cu nanoparticles by tailoring the surface morphologies of TiN-coated electrodes for biosensing applications. *Langmuir* **2013,** *29*, 16025-16033.

(32) Platzman, I.; Brener, R.; Haick, H.; Tannenbaum, R., Oxidation of polycrystalline copper thin films at ambient conditions. *J. Phys. Chem. C* **2008,** *112*, 1101-1108.

(33) Gao, D.; Scholten, F.; Roldan Cuenya, B., Improved CO_2 Electroreduction performance on plasma-activated Cu catalysts via electrolyte design: halide effect. *ACS Catal.* **2017,** *7*, 5112-5120.

(34) Wang, J.-G.; Zhang, Y.; Yu, X.; Hua, X.; Wang, F.; Long, Y.-T.; Zhu, Z., Direct molecular evidence of proton transfer and mass dynamics at the electrode–electrolyte interface. *J. Phys. Chem. Lett.* **2019,** *10*, 251-258.

(35) Zhou, Y.; Su, M.; Yu, X.; Zhang, Y.; Wang, J.-G.; Ren, X.; Cao, R.; Xu, W.; Baer, D. R.; Du, Y.; Borodin, O.; Wang, Y.; Wang, X.-L.; Xu, K.; Xu, Z.; Wang, C.; Zhu, Z., Real-time mass spectrometric characterization of the solid–electrolyte interphase of a lithium-ion battery. *Nat. Nanotechnol.* **2020,** *15*, 224-230.

(36) Labrador, N. Y.; Songcuan, E. L.; De Silva, C.; Chen, H.; Kurdziel, S. J.; Ramachandran, R. K.; Detavernier, C.; Esposito, D. V., Hydrogen evolution at the buried interface between Pt thin films and silicon oxide nanomembranes. *ACS Catal.* **2018,** *8*, 1767-1778.

(37) Esposito, D. V., Membrane-coated electrocatalysts—an alternative approach to achieving stable and tunable electrocatalysis. *ACS Catal.* **2018,** *8*, 457-465.

(38) Feiner, A. S.; McEvoy, A. J., The Nernst Equation. *J. Chem. Educ.* **1994,** *71*, 493.

(39) Barbero, C. A., Ion exchange at the electrode/electrolyte interface studied by probe beam deflection techniques. *Phys. Chem. Chem. Phys.* **2005,** *7*, 1885-1899.

(40) Kempler, P. A.; Coridan, R. H.; Lewis, N. S., Effects of bubbles on the electrochemical behavior of hydrogen-evolving Si microwire arrays oriented against gravity. *Energy Environ. Sci.* **2020,** *13*, 1808-1817.

(41) Compton, R. G., Banks, C. E, *Understanding voltammetry*. World Scientific: 2018.

(42) Alobaid, A.; Wang, C.; Adomaitis, R. A., Mechanism and kinetics of HER and OER on NiFe LDH films in an alkaline electrolyte. *J. Electrochem. Soc.* **2018,** *165*, J3395-J3404.
